# Protective Potentials of Natural Products Against Vascular Dementia: A Comprehensive Review of Experimental Studies

**DOI:** 10.1002/ptr.70448

**Published:** 2026-09-13

**Authors:** Jung‐Hoon Kim, Yongtaek Oh, Suin Cho

**Affiliations:** ^1^ Division of Pharmacology, School of Korean Medicine Pusan National University Yangsan Republic of Korea; ^2^ Department of Diagnostics, College of Korean Medicine Woosuk University Jeonju Republic of Korea

**Keywords:** animal models, chronic cerebral hypoperfusion, molecular signaling pathway, natural products, vascular dementia

## Abstract

Vascular dementia (VaD), accounting for up to 20% of dementia cases globally, is primarily driven by chronic cerebral hypoperfusion (CCH), which leads to progressive cognitive impairment. Despite the increasing prevalence of VaD, effective treatments remain limited. Natural products—including herbal formulas, single herbs, and phytochemicals—have emerged as promising candidates due to their multi‐targeted biological activities. This review aimed to evaluate the efficacy and underlying mechanisms of natural products in ameliorating CCH‐induced VaD in experimental rodent models, and to identify feasible mechanistic pathways and targets. Protective effects of natural products were mediated by enhanced vascular regeneration, BBB stability, synaptic plasticity, and neurotrophic signaling along with reduced oxidative stress, neuroinflammation, and apoptosis. Among the proteins concerned, the serine/threonine kinase Akt1 emerged as a primary regulatory protein involved in coordinating survival and inflammatory responses. Predominantly male rat models were subjected to bilateral common carotid artery occlusion (BCCAo) or bilateral common carotid artery stenosis (BCAS) to mimic clinical features of CCH. These two models provided a robust and translational strategy for a preclinical approach against VaD. The interconnected network analysis facilitated the discovery of core anti‐VaD interventions by linking established herbal formulas with promising, yet understudied, botanical resources. Natural products demonstrate multitargeted protective potential in VaD animal models by targeting multiple pathological processes. Further investigation of underexplored herbs and phytochemicals may expand treatment strategies and support the development of novel and multi‐targeted interventions for VaD.

**Abbreviations**
2VO: two‐vessel occlusion
4VO: four‐vessel occlusion
Aβ: β‐amyloid
AChE: acetylcholinesterase
ADAM10: a disintegrin and metalloprotease 10
AGE: advanced glycation end‐products
AKT: protein kinase B
AMPK: AMP‐activated protein kinase
Ang: angiopoietin
APP: amyloid precursor protein
ATP5A: adenosine triphosphate synthase F1 subunit alpha
BACE1: β‐site APP‐cleaving enzyme 1
Bax: Bcl‐2‐associated X protein
BBB: blood–brain barrier
BCAS: bilateral common carotid artery stenosis
BCCAo: bilateral common carotid artery occlusion
Bcl‐2: B cell lymphoma‐2
BDNF: brain‐derived neurotrophic factor
CaMKII‐α: calcium/calmodulin‐dependent protein kinase II alpha
cAMP: cyclic adenosine monophosphate
CAT: catalase
CCH: chronic cerebral hypoperfusion
CDC20: cell division cycle 20
C/EBPβ: CCAAT/enhancer binding protein beta
ChAT: choline acetyltransferase
CREB: cAMP response element‐binding protein
CRMP2: collapsin response mediator protein‐2
CYP: cytochrome P450
EGF: epidermal growth factor
eNOS: endothelial nitric oxide synthase
EPO: erythropoietin
ER: endoplasmic reticulum
ErbB4: Erb‐b2 receptor tyrosine kinase 4
ERK: extracellular signal‐regulated kinase
FoxO: Forkhead box O
GABA: γ‐aminobutyric acid
GAP‐43: growth‐associated protein 43
GLUT: glucose transporter
GO: gene ontology
GPR: G protein‐coupled estrogen receptor
GPX: glutathione peroxidase
GSH: glutathione
GSK‐3β: glycogen synthase kinase‐3β
HDAC2: histone deacetylase 2
HIF‐1: hypoxia‐inducible factor‐1
HO‐1: heme oxygenase 1
HUMMR: hypoxia‐upregulated mitochondrial movement regulator
IDE: insulin‐degrading enzyme
IGF‐1: insulin‐like growth factor‐1
IL: interleukin
iNOS: inducible nitric oxide synthase
JAK: Janus kinase
Keap1: Kelch‐like ECH associated protein 1
KEGG: Kyoto encyclopedia of genes and genomes
LDH: lactate dehydrogenase
LINGO1: leucine rich repeat and Ig domain containing 1
LPS: lipopolysaccharide
LXRα: liver X receptor alpha
MAP‐2: microtubule‐associated protein‐2
MAPK: mitogen‐activated protein kinase
MCP‐1: monocyte chemoattractant protein‐1
MDA: malondialdehyde
MECP2: methyl CpG binding protein 2
MTCO1: mitochondrially encoded cytochrome c oxidase subunit 1
MYD88: myeloid differentiation primary response 88
NeuN: neuronal nuclei
NF: neurofilament
NF‐κB: nuclear factor kappa B
NGF: nerve growth factor
NMDA: N‐methyl‐D‐aspartic acid
NLRP3: nucleotide oligomerization domain‐like receptors 3
NR2A: N‐methyl‐D‐aspartate receptor 2A
Nrf2: nuclear factor‐E2‐related factor 2
NRG‐1: neuregulin‐1
NSE: neuron‐specific enolase
NVU: neurovascular unit
PI3K: phosphatidylinositol‐3‐kinase
PINK1: phosphatase and tensin homolog‐induced kinase 1
PKA: protein kinase A
PP2A: protein phosphatase 2A
PPAR‐γ: peroxisome proliferator‐activated receptor‐gamma
PPI: protein–protein interaction
PSD: postsynaptic density protein
RAGE: receptor for AGE
ROCK2: Rho associated coiled‐coil containing protein kinase 2
ROS: reactive oxygen species
SIRT3: Sirtuin 3
SMAD: suppressor of mothers against decapentaplegic
SOD: superoxide dismutase
STAT: signal transducers and activators of transcription
TFAM: transcription factor A mitochondrial
TGF‐β: transforming growth factor‐β
Tie‐2: tunica interna endothelial cell kinase‐2
TLR4: toll‐like receptor 4
TNF: tumor necrosis factor
TRAF1: TNF receptor‐associated factor 1
TRAK2: trafficking protein kinesin 2
TREM2: triggering receptor expressed on myeloid cells 2
TrkB: tropomyosin receptor kinase B
TRPM: transient receptor potential melastatin
UCCAo: unilateral common carotid artery occlusion
VaD: vascular dementia
VAChT: vesicular acetylcholine transporter
VEGF: vascular endothelial growth factor
ZO‐1: zona occludens‐1

## 1 Introduction

Vascular dementia (VaD), the second most prevalent dementia type, is primarily caused by the impaired blood supply to the brain, resulting in progressive cognitive and behavioral deterioration (Plassman et al. 2007). Cardiovascular disorders such as ischemic/hemorrhagic stroke, hypertension, diabetes mellitus, or hyperlipidemia can affect the onset of VaD (Inoue et al. 2023). Diabetes, particularly with early onset, doubles the risk of dementia and is linked to microvascular damage, neuroinflammation, and cerebral atrophy. Hypertension in midlife, if untreated, increases VaD risk later through white matter damage and vascular changes, whereas metabolic syndromes, such as obesity and insulin resistance, accelerate cognitive decline, particularly before the age of 70 (Venkat et al. 2015). People with VaD often present various symptoms, including memory decline, cognitive deficits, confusion, and even behavioral dysfunction (Day et al. 2022). The progression of these symptoms can be either stepwise—with noticeable declines after a certain disease—or gradual, depending on the nature and extent of the blood flow issues. Chronic cerebral hypoperfusion (CCH) is characterized by prolonged, insufficient blood flow to the brain over weeks, months, or even years. Progressive narrowing or obstruction of cerebral arterioles, arterial stiffening, or sustained vascular hypertension can disrupt cerebral perfusion, ultimately impairing brain function (Wolters et al. 2017). Reduced cerebrovascular blood flow to the brain is a major contributor to progressive cognitive decline and memory impairment accompanied by neuronal loss, synaptic dysfunction, or neuroinflammation (Chai et al. 2023). These CCH‐induced pathological changes in the brain are apparently associated with VaD progression. Moreover, VaD results from various pathophysiological causes, such as major strokes affecting cognitive regions or silent microvascular strokes linked to small vessel disease, which causes lacunar infarcts and ischemic white matter lesions. These strokes and vessel damage ultimately induce cognitive decline, the hallmark of VaD (Venkat et al. 2015; Tiwari et al. 2021).

VaD rodent models are efficient for studying vascular abnormalities and cognitive impairments. Vessel occlusion models, including bilateral common carotid artery occlusion (BCCAo), unilateral common carotid artery occlusion (UCCAo), and bilateral common carotid artery stenosis (BCAS), mimic hypoperfusion and ischemic conditions. Risk factor‐based models address hypertension, diabetes, hyperhomocysteinemia, and age‐related issues. Other models include muscarinic‐5 receptor transgenic mice, cerebral autosomal dominant arteriopathy with subcortical infarcts and leukoencephalopathy, cerebral amyloid angiopathy, high fat diet‐induced models, and multiple‐infarct models. Vessel occlusion models stand out due to their cost‐effectiveness, easy recovery from surgery, and ability to induce predictable cognitive impairment. Of the abovementioned occlusion models, BCAS is particularly promising for chronic hypoperfusion‐related VaD, as it replicates key features, such as white matter damage and gliosis, and is suitable for genetically modified animal studies. These vascular occlusion models advance molecular and physiological research, aiding in the evaluation of treatments and understanding VaD mechanisms (Tiwari et al. 2021).

Efforts to develop the options for VaD treatment have focused on creating effective animal models that closely mimic human conditions, as VaD often lacks effective conventional therapies and no drugs are currently approved by the FDA specifically to treat VaD. Therefore, research on the VaD rodent models has explored the potential of natural products as alternative treatments. Studies have highlighted the benefits of herbs with antioxidant, anti‐inflammatory, and neuroprotective properties for preventing VaD. For instance, *Scutellaria baicalensis* extracts mitigated spatial memory impairments, hippocampal mitogen‐activated protein kinase (MAPK) signaling alterations, and microglial activation in CCH‐ or lipopolysaccharide‐induced rats (Hwang et al. 2011). Herbal formulas, such as Cardiotonic Pill and Danggui Shaoyao San, improved cognitive abilities and daily functioning (Lee et al. 2013; Yuan et al. 2024). In addition, icariin, a phytochemical from *Epimedium brevicornu*, improved learning and memory, reduced oxidative stress, and preserved cholinergic system function in a CCH‐induced cognitive deficit rat model (Xu et al. 2009). Although these findings are promising, many studies face methodological limitations, and further research is needed to establish the efficacy and safety of these treatments. Natural products offer hope for managing VaD, thus emphasizing the importance of integrating traditional knowledge with modern methodological approaches to develop effective therapies for this challenging condition.

## 2 Methodology

### 2.1 Selection of Literatures

The research papers published from Jan 1st 2000 to Dec 31st 2024 were collected from PubMed and Google Scholar using the search terms; “vascular dementia,” “chronic cerebral hypoperfusion,” “herb,” “herbal medicine,” and “natural product.” The papers were included in this study if they met the following criteria: (1) original research papers, (2) in vivo experimental studies, (3) studies using herbal formulas, single herbs, and phytochemicals, (4) animal models of VaD and CCH, and (5) research papers published in English. The exclusion criteria were as follows: (1) clinical study, (2) exclusively in vitro experimental studies, (3) non‐experimental studies (exclusively network pharmacology and review studies), (4) animal models of cerebral diseases other than VaD and CCH (e.g., Alzheimer disease, stroke, or ischemic brain injury), (5) studies using non‐herbal materials or non‐phytochemicals, and (6) non‐English research papers.

### 2.2 Selection of Methodology of CCH Animal Models

In developing this review, all preclinical literature on rodent models of VaD was comprehensively analyzed. Among the many methodologies described, such as BCCAo (or two‐vessel occlusion, 2VO), BCAS, four‐vessel occlusion (4VO), UCCAo, and micro‐embolic and metabolic risk factor models, the BCCAo and BCAS protocols emerged as the two most frequently employed and best characterized methods for inducing CCH in animal models (Venkat et al. 2015; Tiwari et al. 2021; Hattori et al. 2016). We therefore restricted our detailed analysis to these two models, both for their high usage across laboratories and for their complementary strengths in recapitulating human subcortical ischemic and global hypoperfusion syndromes.

### 2.3 PPI/GO/KEGG and Network Analyses

A protein–protein interaction (PPI) network was constructed using quantified protein data and gene expression levels derived from Western blot and PCR results in previous studies. The identified proteins were entered into the STRING (https://string‐db.org) database to analyze their interactions, and Cytoscape (ver. 3.10.3) software was used for visualization. Additionally, Gene Ontology (GO) information and KEGG pathway analysis provided by the STRING database were incorporated to clearly illustrate key proteins regulated by herbal resources and their mechanisms of action.

The target protein datasets used for PPI and functional enrichment analyses were systematically curated from the experimental findings identified through our review. To ensure complete transparency and reproducibility, these curated targets have been structured into an integrated master mapping table provided as Table S1. This table establishes a clear trajectory from individual primary references to the bioinformatic workflow, categorized by herbal formulas, single herbs, and phytochemicals. Targets were eligible for inclusion if they were verified via direct experimental measurements (such as Western blot or RT‐PCR) in the cited literature. Specifically, only targets demonstrating statistically significant alterations (*p* < 0.05) in protein or gene expression following natural product intervention were incorporated into the final dataset, whereas non‐significant findings were excluded from PPI network construction and GO/KEGG enrichment analyses. During data curation, functional isoforms and post‐translationally modified or activated forms (e.g., phosphorylated kinase cascades and cleaved caspases) were merged under their representative canonical gene symbols to maintain network topographical integrity and prevent artificial node inflation.

To construct the herb network, a binary herbal formula–herbal ingredient matrix was first generated to represent a 2‐mode network. This bipartite network was then projected into a 1‐mode herb–herb network by linking herbal ingredients that co‐occurred within the same herbal formula. The edge weights between herbal ingredients indicate the frequency of their co‐occurrence across herbal formulas. To remove rare and potentially spurious co‐occurrences and to focus on meaningful relationships between herbal ingredients, the network was filtered to retain only herb pairs that co‐occurred at least twice (i.e., edge weight ≥ 2). This threshold was not determined through formal statistical optimization but was adopted as an exploratory and pragmatic choice. After applying the edge‐weight threshold, the final filtered herb–herb network consisted of 35 nodes and 224 edges. Community detection was subsequently conducted using the Clauset–Newman–Moore (CNM) algorithm, a hierarchical agglomerative method that identifies community structures by maximizing modularity. The CNM community detection yielded a modularity score of 0.336057, and four communities (G1–G4) were ultimately identified. All network analyses and visualizations were performed using NetMiner (Version 4.5.1.e, 2025; Cyram Inc., Seoul, Korea).

## 3 Key Pathological Pathways of VaD Inhibited by Natural Products

Tables S2–S4 list the natural products, including herbal formulas, single herbs, and phytochemicals that exhibited protective effects in VaD animal models. These natural products are categorized by the molecular signaling pathways through which they exert inhibitory effects against the key pathological mechanisms.

### 3.1 Vascular Injury and Hypoperfusion

The animal model of VaD is established by reduced cerebral blood flow due to damaged or narrowed vessels. This leads to chronic hypoperfusion, depriving the brain tissue of oxygen and nutrients. Molecular markers relating to vascular repair and angiogenesis, such as VEGF, Ang‐1/2, Tie‐2, and eNOS, are downregulated, whereas markers like GLUT1/3 and LDH are increasingly expressed as a sign of energy metabolism dysfunction under oxygen‐deprived conditions (Gao et al. 2025; Garrett and Niccoli 2022).

Modified Dioscorea Pill enhanced vascular stability and regeneration by upregulating Ang‐1, Ang‐2, Tie‐2, and VEGF, promoting neuroplasticity, and restoring neurovascular unit integrity through the upregulation of GAP‐43 and VEGF gene expression in the hippocampus of male VaD rats at a single‐dose level (Kuang et al. 2023; Li et al. 2020; Li 2019). Ginseng Radix ( *Panax ginseng* root) ameliorated learning and memory deficits in a single behavioral test (Morris water maze) and promoted cholinergic activity and angiogenesis, thereby protecting against vascular damage and suppressing neuroapoptosis (Zhu et al. 2018).

### 3.2 BBB Breakdown and Neuroinflammation

Hypoxia causes endothelial dysfunction and breakdown of the BBB, reflected by the downregulation of tight junction‐ and BBB integrity‐related markers, such as claudin‐5, occludin, and ZO‐1 (Hashimoto et al. 2021). BBB disruption allows the infiltration of inflammatory cells and cytokines (IL‐1β, IL‐6, and TNF‐α) into the brain, triggering neuroinflammation with increased microglial markers (CD68, CD16) (Wang, Cao, et al. 2018; Graves and Baker 2020).

Tongqiao Yizhi Granule inhibited pyroptosis and related inflammatory responses by activating CREB in male VaD rat hippocampus (Li, Hao, et al. 2024; Chen, Tang, et al. 2024). The herbal combination (WCJ) of Tritici Fructus Levis (wheat bran), Citri Reticulatae Pericarpium (citrus peel), and Jujubae Fructus (jujube) inhibited glial activation in the damaged white matter of male VaD rats (Kim, Lim, Hwang, and Lee 2024). Banhabaekchulcheonma‐tang protected against cerebral ischemia‐induced cortex injury in male mice by regulating the expression of CDC20, EGF, and TRAF1, and related neuroactive proteins (Kim, Lim, Cho, and Kim 2024). Buqi Huoxue Tongnao Prescription, Modified Buyang Huanwu Decoction, and Modified Dioscorea Pill prescription alleviated neuroinflammation, neuroapoptosis, and gliosis in male VaD rat hippocampus (Li 2019; Gao et al. 2024; Yang et al. 2021). Zishen Huoxue Decoction Prescription Granule relieved the NVU‐injured NLRP3 inflammatory cascade and pyroptosis by upregulating miR‐124‐3p, but the doses were not specified (Yao et al. 2023). The Xi Xian Tong Shuan Capsule and Nao Tai Fang protected against myelin damage and neuronal loss, and suppressed proinflammatory factors (such as IL‐6, IL‐1β, and TNF‐α) in the white matter and the hippocampus of CCH‐induced male rats at a single dose level (Yan et al. 2022; Zhao et al. 2021). Hwangryunhaedok Tang exhibited memory enhancement in Y‐maze test and novel object recognition test, cholinergic activation, and anti‐inflammatory effects by inhibiting p38 MAPK phosphorylation in male VaD rat hippocampus (Sohn et al. 2019). Kaixin Jieyu Prescription increased neurogenesis, restored the fibrinolytic system, and reduced hyper‐permeability and inflammation in the BBB of male VaD rats (Pan et al. 2015). Cardiotonic Pill attenuated the structural damage and inflammatory reaction in the white matter and the hippocampus of male VaD rats by inhibiting the MAPK signaling pathway at a single dose level (Lee et al. 2013).

Gardeniae Fructus ( *Gardenia jasminoides* fruit) inactivated microglia by activating the PPAR‐γ signaling pathway, inhibiting NF‐κB, and elevating C/EBPβ signaling in male VaD rats (Liu et al. 2022). Chuanxiong Rhizoma (*Ligusticum chuanxiong* rhizome) also attenuated cognitive impairment in a single behavioral test (novel object recognition) and the resulting pathological damage by suppressing MAPK signaling‐related proteins (such as ERK and p38) in the white matter around the hippocampus in male VaD mice (Lim et al. 2024). Pretreatment with Perillae Folium ( *Perilla frutescens* leaf) extract recovered memory deficits, neuronal damage, and microglial activation in male VaD rats (Kang et al. 2022). Epimedii Herba improved cognitive function in a single behavioral test (Morris water maze), inhibited the activation of microglia and astrocytes, and alleviated neuroinflammation by suppressing TNF signaling pathways in the hippocampus of male VaD mice (Xie et al. 2022). Broussonetiae Fructus ( *Broussonetia papyrifera* fruit) led to cognitive improvement in a single behavioral test (Morris water maze) and lowered microglial activation by inhibiting M1 polarization—the decreased iNOS and Fc fragment of IgG receptor IIIa proteins—and facilitating M2 polarization—the increased arginase‐1 protein—in male VaD rats, but the dose was not specified (Liu, Wang, et al. 2020). Ginkgo Folium ( *Ginkgo biloba* leaf) and Mume Fructus ( *Prunus mume* fruit) reduced glial proliferation, astrocytic activation, neuroinflammatory cytokines (COX‐2, IL‐1β, IL‐6, TLR4, p‐ERK, and NF‐κB), and p38 MAPK activity but activated the cholinergic response by increasing choline acetyltransferase in male CCH rats (Kim et al. 2016a, 2016b; Lee et al. 2015; Jeon et al. 2012). Salviae Miltiorrhizae Radix et Rhizoma ( *Salvia miltiorrhiza* root and rhizome) protected against neuronal damage caused by microglial activation and neuroinflammation by downregulating MyD88‐dependent TLR4 signaling pathways in the white matter and hippocampus of male VaD rats (Kim et al. 2015). Scutellariae Radix (*Scutellaria baicalensis* root) attenuated spatial memory impairments and hippocampal MAPK signaling in male VaD rats (Hwang et al. 2011).

Gastrodin, a phenolic glucoside from *Gastrodia elata*, downregulated the p38 MAPK signaling pathway in male VaD rat hippocampus (Liu et al. 2018). Mumefural, a furaldehyde from the heat‐processed *Prunus mume* fruit, improved cognitive function in a single behavioral test (Morris water maze) by increasing synaptic markers and cognition‐related proteins, and suppressed cholinergic dysfunction and neuronal loss, neuroinflammation, and gliosis by inhibiting TLR4/MyD88, NLRP3, and NF‐κB signaling pathways in the basal forebrain and hippocampus of male VaD rats (Bang et al. 2019). Cornel iridoid glycoside (morroniside and loganin at a ratio of 2.3:1) from *Cornus officinalis* protected against neuroinflammation by downregulating the NLRP3/calpain pathway in male VaD rats (Zheng et al. 2022). Rehmannioside A, an iridoid glucoside from *Rehmannia glutinosa*, protected against cognitive dysfunction and suppressed oxidative stress, neuroinflammation, and apoptosis by activating Nrf2 and inactivating NF‐κB and caspase‐3 in the hippocampus of male VaD rats (Sun et al. 2019). Tanshinol, a phenolic acid from *Salvia miltiorrhiza* , and tetrandrine, a bisbenzylisoquinoline alkaloid from *Stephania tetrandra*, protected against neuroinflammatory and necrotic damage by inhibiting IL‐1β, IL‐6, TNF‐α, iNOS, N‐methyl‐D‐aspartate receptor 2B phosphorylation, and astrocyte‐derived S100B in the hippocampus of male VaD rats (Yang et al. 2016; Lv et al. 2016; Chen et al. 2013).

### 3.3 Oxidative Stress and Mitochondrial Dysfunction

Inflammation generates reactive oxygen species (ROS), leading to cellular oxidative damage by upregulating 4‐hydroxynonenal and 8‐hydroxy‐2′‐deoxyguanosine, and mitochondrial dysfunction by downregulating mitochondrial markers (ATP5A, TFAM, and MT‐CO1) (Shang et al. 2019).

Zishen Huoxue Decoction Prescription Granule restored mitochondrial structure and function, alleviated oxidative stress and neuroapoptosis, and improved mitophagy via the PINK1/Parkin signaling pathway in male VaD rat hippocampus (Zhao et al. 2024). Modified Daehwang Hwanglyoun Sasim Tang and Danshen Honghua herbal pair improved learning and memory function and prevented neuronal damage via cholinergic enhancement and antioxidant activity induction in male VaD rat hippocampus at a single dose level (Lee et al. 2022; Zhang et al. 2022). Yi Gan San Granule facilitated spatial learning and memory abilities in a single behavioral test (Morris water maze) by modulating the metabolism of arachidonic acid, glycerophospholipid, tryptophan, and sphingolipid in male VaD rats given a single dose level (Liao et al. 2020). Dengzhan Shengmai Capsule and its active components exerted neuroprotective effects in male VaD rats by increasing GABA_A_ receptor activation and attenuating NMDA receptor overactivation (Sheng et al. 2020). Choto San triggered spatial learning improvement and Nrf2‐mediated antioxidant response (Jiang et al. 2019), upregulated M‐CSF mRNA expression in the brain (Obi et al. 2007), and activated cholinergic stimulation through the muscarinic M_1_ receptor, with a considerable contribution of Uncaria, a crucial herb in Choto San, in VaD animal models (Zhao et al. 2007; Murakami et al. 2005). Daming Capsule suppressed the oxidative stress markers such as MDA and enhanced antioxidant enzymes such as SOD in male VaD rats (Song et al. 2015). Bushen Huoxue Decoction (a combination of Epimedii Herba and Salviae Miltiorrhizae Radix et Rhizoma) showed antioxidant‐mediated cognitive enhancement, but the information on the sex of rats was not specified (Ye et al. 2014). Gardeniae Fructus increased myelin‐related proteins, improved lipid metabolism, and lowered oxidative stress by activating the Keap1‐Nrf2/HO‐1, sphingolipid, and glycerophospholipid metabolic pathways in VaD mice (Pang et al. 2024). *Ginkgo biloba* extract recovered neuronal loss and behavioral impairment and increased anti‐oxidative reaction in male VaD gerbils (Rocher et al. 2011). Gastrodin ameliorated cognitive deficits in a single behavioral test (Morris water maze), neurodegenerative injuries, and mitochondrial dysfunction‐related oxidative stress by upregulating the SIRT3/TFAM acetylation/oxidative phosphorylation/mitochondrial function in male VaD rats (Chen, Yang, et al. 2024). Gastrodin also suppressed hippocampal neuronal ferroptosis, reduced iron deposition and lipid peroxidation, and enhanced the antioxidant effect via the Nrf2/Keap1‐GPx4 signaling pathway in male VaD rats (Li et al. 2022). Salidroside, a phenylpropanoid glycoside from *Rhodiola rosea* , ameliorated hippocampal oxidative stress in male VaD rats (Tian et al. 2020). Glycyrrhizin (glycyrrhizic acid), a triterpene glycoside from *Glycyrrhiza* sp., inhibited oxidative stress and reduced the current amplitude of voltage‐gated sodium channels by decreasing cytochrome‐c and increasing GSH in male VaD rats at a single dose level (Sathyamoorthy et al. 2020; Guo et al. 2016; Hou et al. 2010). Betaine, an amino acid derivative from either *Lycium chinense* or *L. barbarum* , exhibited anti‐oxidative reactions in male VaD rat hippocampus at a single dose level over a short period (Nie et al. 2016). Bilobalide, a sesquiterpene trilactone from *Ginkgo biloba* , suppressed neuroapoptosis, oxidative damage, and TNF‐α in the cortex and the hippocampus of male VaD rats (Li et al. 2013). Polydatin (a stilbene glucoside from *Reynoutria japonica* ) and crocin (a carotenoid from *Crocus sativus* ) showed cognitive and behavioral enhancement in a single behavioral test (Morris water maze) and anti‐oxidative effect in male VaD rats (Li et al. 2012; Hosseinzadeh et al. 2012).

### 3.4 Amyloid Accumulation and Proteostasis Failure

Impaired clearance of damaged synaptic components across a damaged BBB also contributes to abnormal accumulation of Aβ peptides (Aβ_40/42_) and hinders their degradation by downregulating ADAM10 and IDE (Ullah and Lee 2023).

Xiaoshuan Enteric‐Coated Capsule protected against cognitive impairment in male VaD rats by regulating the AKT/GSK‐3β/CRMP2 signal pathway (Li et al. 2018). The extract of *Scutellaria baicalensis* leaf enhanced cognitive function and potentially suppressed tau phosphorylation by downregulating kinase‐mediated phosphorylation and upregulating PP2A‐catalyzed dephosphorylation in the cortex and hippocampus of ischemia‐induced male VaD rats (Cao et al. 2016). Gastrodin and gastrodigenin exhibited neuroprotection against memory impairments, neuronal death, brain energy metabolism disorders, and Aβ deposition by downregulating Aβ deposition and tau activation in male VaD rat hippocampus (Shi et al. 2020; Wu et al. 2023). Osthole, a coumarin from *Cnidium monnieri* , attenuated NLRP3 inflammasome‐mediated Aβ deposition in male VaD rat hippocampus (Liu, Chen, et al. 2020). Scutellarin, a flavone glucuronide from *Scutellaria baicalensis*, reduced learning and memory impairment in a single behavioral test (Morris water maze), microglial activation, and Aβ formation by inhibiting APP and BACE‐1 expressions in male VaD rat hippocampus (Shin et al. 2018). Tanshinone IIA, a diterpene quinone from *Salvia miltiorrhiza* , protected against memory and behavioral damage, activated the cholinergic system, and attenuated tau phosphorylation by suppressing GSK‐3β protein in the hippocampus of male VaD rats at a single dose level over a short period (Kong et al. 2017). Icariin, a prenylated flavonol glycoside from *Epimedium brevicornu*, attenuated cognitive dysfunction in a single behavioral test (Morris water maze), reduced Aβ production via BACE1 downregulation and ADAM10 upregulation, and promoted Aβ degradation by upregulating IDE, which involved TGF‐β signaling pathway, cholinergic system, and oxygen free radical scavenging in male VaD rats (Xu et al. 2009; Li et al. 2015).

### 3.5 Autophagy Impairment and ER Stress

Chronic stress‐induced ROS overproduction damages cerebral proteins and organelles, subsequently disrupting autophagic pathways and the cellular system for eliminating damaged components (Wang, Zhang, et al. 2020). The stress, caused by an overloaded endoplasmic reticulum and failed protein folding, activates PERK, IRE1α, and ATF6, accompanied by eIF2α phosphorylation and ATF4 upregulation (Roshdy et al. 2024).

Bushen Huoxue Formula mitigated nerve cell deterioration and cognitive deficit by reducing excessive autophagy in male VaD rat hippocampus at a single dose level (Luo et al. 2021). Taohong Siwu Decoction downregulated p‐PERK, p‐IRE1α, eIF2α, ATF6, and ATF4 proteins in male VaD rat hippocampus (Fan et al. 2024). Gastrodin inhibited excessive autophagy and apoptosis by activating the PI3K/AKT pathway in male VaD rat hippocampus (Chen et al. 2023).

### 3.6 Synaptic Dysfunction and Neurotransmission Failure

Sustained neuroinflammation, oxidative stress, and amyloid toxicity reduce synaptic proteins (synapsin‐1, PSD95, and GAP‐43), leading to synaptic plasticity damage and downregulated synaptic plasticity pathways (CaMKII/CREB) (Wang, Cao, et al. 2018; Zhao et al. 2004). Acetylcholinesterase upregulation and ChAT/VAChT downregulation impair the cholinergic signaling pathway, and the overactivation of NMDA receptor subunits (NR2B) causes excitotoxic calcium influx and neuronal damage (Wang et al. 2009).

Tongqiao Huoxue Decoction enhanced cognitive ability and synaptic remodeling in neuronal damage in VaD rat hippocampus via cAMP/PKA/CREB signaling activation (Li, Ye, et al. 2024) and suppressed excessive intracellular calcium and hyperphosphorylation of PKA, CaMKII, and CREB in both male and female VaD rats (Ge et al. 2015). Tongqiao Yizhi Granule also inactivated the NF‐κB/NLRP3/caspase‐1 pathway and enhanced the plasticity of synaptic structures, the synaptic transmission, and learning and memory in a single behavioral test (Morris water maze) via the MAPK/ERK signaling cascade in male VaD rat hippocampus (Li, Hao, et al. 2024; Chen, Tang, et al. 2024). Buqi Huoxue Tongnao Prescription exerted a protective effect against lipidemia‐related damage in male CCH rats by reducing neuroinflammation, apoptosis, oxidative stress, and lipidemia via PI3K/AKT and LXRα/CYP7A1 signaling activation (Gao et al. 2024). Shenzhi Jiannao Formula attenuated cognitive deficits accompanied by neuronal injury and glutamate‐induced cytotoxicity by regulating glutamate synaptic vesicle transport and the internalization of NMDA receptors in male VaD rat hippocampus (Tian et al. 2021). The flavonoids from Epimedii Herba also ameliorated cognitive deficits, inhibited neuronal apoptosis, and enhanced synaptic plasticity by activating the NRG‐1/p‐ErbB4, BDNF/p‐Fyn, and PI3K/Akt/CREB signaling pathways in male VaD rat hippocampus (Niu, Ma, et al. 2020). Schizandrin B, a lignan from *Schisandra chinensis*, ameliorated cognitive impairment in a single behavioral test (Morris water maze), hippocampal cell damage, and neuronal loss in the hippocampus by reducing HIF‐1α, TRAK2, and HUMMR in male VaD rats (He et al. 2024). Puerarin, an isoflavone from *Pueraria montana var. lobata* , enhanced learning in a single behavioral test (Morris water maze) and ameliorated memory dysfunction, hippocampal neurodamage, spine alterations, and apoptosis via TRPM2 suppression and NR2A induction via the NMDAR pathway, and also mitigated nerve injury and protected hippocampal neurons by increasing pMeCP2 in male VaD rats (Zhu et al. 2021; Wang, Zhang, et al. 2018). Betaine improved cognition and restored synaptic integrity by upregulating PSD93, PSD95, and MAP2 proteins in male VaD rats at a single dose level over a short period (Nie et al. 2016).

### 3.7 Neuronal Apoptosis and Senescence

Oxidative stress, synaptic integrity loss, energy failure, and inflammation ultimately trigger apoptotic signaling by causing an increased Bax/Bcl‐2 ratio and inducing cytochrome c‐mediated caspase‐3 activation (Zhong et al. 2024). DNA fragmentation and cytoskeletal damage (spectrin αII cleavage) lead to marked progressive neurodegeneration. The upregulation of cellular aging markers (p16, p21, and p53) leads to irreversible neuronal loss (Graves and Baker 2020).

Zishen Huoxue Decoction Prescription Granule also restored mitochondrial structure and function, alleviating oxidative stress, neuronal apoptosis, and mitophagy via the PINK1/Parkin signaling pathway in male VaD rat hippocampus (Zhao et al. 2024). Danggui Shaoyao San alleviated neuronal apoptosis and improved synaptic plasticity in male VaD rat hippocampus via HIF‐1α/EPO pathway activation (Yuan et al. 2024). Taohong Siwu Decoction also mitigated pathological changes in male VaD rat hippocampus by suppressing the ER‐mediated apoptosis signaling pathway (Fan et al. 2024). Modified Erchen Decoction inhibited the JAK2/STAT3 and JNK/BAX signaling pathways in male VaD rats (Gao et al. 2023). Modified Buyang Huanwu Decoction enhanced cognitive ability in a single behavioral test (Morris water maze) and suppressed oxidative stress, inflammation, and apoptosis in male VaD rats (Yang et al. 2021). Honokiol, a lignan from *Magnolia officinalis*, inhibited astrocyte A1 polarization and toxicity via SIRT3‐dependent inhibition of STAT3 in male CCH rats (Hu et al. 2023). Ligustilide, a dihydrophthalide from *Angelica sinensis*, improved cognition in a single behavioral test (Morris water maze) and provided neuroprotection by inhibiting neuroapoptosis, dendritic damage, and astrocyte activation via AMPK/SIRT1 signaling in the cortex and the hippocampus of male VaD rats (Peng et al. 2022; Feng et al. 2012). Curcumin, a diarylheptanoid from *Curcuma longa* , improved cognition in a single behavioral test (Morris water maze) and reduced hippocampal neuroapoptosis by suppressing microglial activation, TREM2/TLR4/NF‐κB pathways, and NLRP3‐dependent pyroptosis in male VaD rats induced by CCH combined with diabetes mellitus (Zheng et al. 2021). Salidroside enhanced cognitive function in a single behavioral test (Morris water maze) and reduced caspase‐3‐dependent apoptosis by suppressing the p38 MAPK signaling pathway in male VaD rats (Tian et al. 2020; Yan et al. 2015). Glycyrrhizin and osthole improved learning and behavioral deficits, increased pyramidal neurons, and reduced mitochondrial‐mediated neuronal damage, microglial activation, oxidative stress, and neuronal apoptosis (Sathyamoorthy et al. 2020; Ji et al. 2010). Ligustrazine, a tetramethylpyrazine from *Ligusticum chuanxiong*, regulated proapoptotic and antiapoptotic biomarkers by promoting BDNF and inactivating MCP‐1 and homocysteine in male VaD rat hippocampus (Zhao et al. 2018).

### 3.8 Neurotrophic Signaling Failure

Endothelial dysfunction and BBB disruption reduce the neurotrophic factors essential for neuronal growth and survival. Inflammation disrupts BDNF/TrkB signaling, leading to reduced neurogenesis and impaired synaptic maintenance. Impaired PI3K/AKT and MAPK/ERK signaling weaken survival pathways. HDAC2 and MECP2 dysregulation suppress neuroprotective gene expression, thus exacerbating neuronal dysfunction. GSK‐3β activation promotes tau hyperphosphorylation and neurodegeneration (Song 2024; Gao et al. 2022). Wnt/β‐Catenin and TGF‐β/SMAD dysregulation contribute to defective repair and fibrosis (Hussain et al. 2022).

Angelicae Sinensis Radix (*Angelica sinensis* root) improved cognition in a single behavioral test (Morris water maze) and promoted neurogenesis in CCH‐induced male VaD rats by increasing BDNF, p‐CREB, GABA, and NGF expression (Xin et al. 2013; Zheng et al. 2008). Mumefural exhibited beneficial effects on memory impairments via ERK/CREB/BDNF signaling activation in male VaD mice at a single dose level (Kim et al. 2023). Ginsenoside Rg_1_ and Rd., triterpenoids from *Panax ginseng* , prevented cognitive injury and neuronal apoptosis by promoting GPR30 expression and BDNF signaling via increased H3 acetylation and HDAC2 levels in the hippocampus of CCH‐induced male VaD mice (Shen et al. 2021; Wan et al. 2017). Andrographolide, a labdane diterpenoid from *Andrographis paniculata* , protected against inflammation‐mediated hippocampal neuronal damage by upregulating the BDNF–TrkB signaling pathway and ameliorated cognitive dysfunction in a single behavioral test (Morris water maze) in male VaD rats (Wang et al. 2019). Salvianolic acid B, a phenolic acid from *Salvia miltiorrhiza* , enhanced cognitive function by upregulating IGF‐1 and p‐Akt in male VaD rats at a single dose level (Ma et al. 2017).

### 3.9 Neuronal Damage and VaD

Neuronal loss and reduced levels of neurotrophic factors, particularly in the hippocampus and the cortex, induce neuronal integrity impairment (MAP‐2, NeuN, NF‐200, and spectrin αII downregulation) and neuronal injury (NSE upregulation) (de Wolf et al. 2020). Consequently, these factors accelerate memory loss, executive dysfunction, and cognitive decline, leading to exacerbated dementia symptoms.

Epimedii Herba flavonoids alleviated fiber bundle and myelin damage and oligodendrocyte loss by inhibiting Lingo‐1/Fyn/ROCK signaling pathway and activating BDNF/TrkB, NRG1/ErbB4, and PI3K/Akt/CREB signaling pathways in the white matter of male VaD rats (Niu, Wang, et al. 2020). Glycosides from *Cistanche* sp. improved learning and memory by reducing tau protein (Chen et al. 2015). Glycyrrhizin impeded GSK‐3β activity by suppressing tau hyperphosphorylation in male VaD rats at a single dose level (Sathyamoorthy et al. 2020). Paeoniflorin, a monoterpenoid glycoside from *Paeonia* sp., protected against neuronal apoptosis and decreased NSE and S100β in male VaD rat hippocampus (Zhang et al. 2016).

Figure 1 illustrates the stepwise protective mechanisms of natural products on VaD animal models, which: (1) increase angiogenic and metabolic support markers and reduce stress signals, (2) stabilize BBB by modulating proinflammatory signals and anti‐inflammatory cytokines, (3) enhance antioxidant systems by upregulating antioxidants such as SOD, CAT, GPx, GSH, and Nrf2, and energy‐producing mitochondrial function, (4) promote proteostasis by reducing Aβ production and enhancing clearance, (5) restore autophagy and alleviate ER stress, aiding cellular homeostasis, (6) boost synaptic plasticity by modulating CREB/BDNF and cholinergic pathways, (7) inhibit apoptosis pathways and promote neuron survival, and (8) improve neurotrophic signaling, reversing some neurodegenerative processes.

**FIGURE 1:**
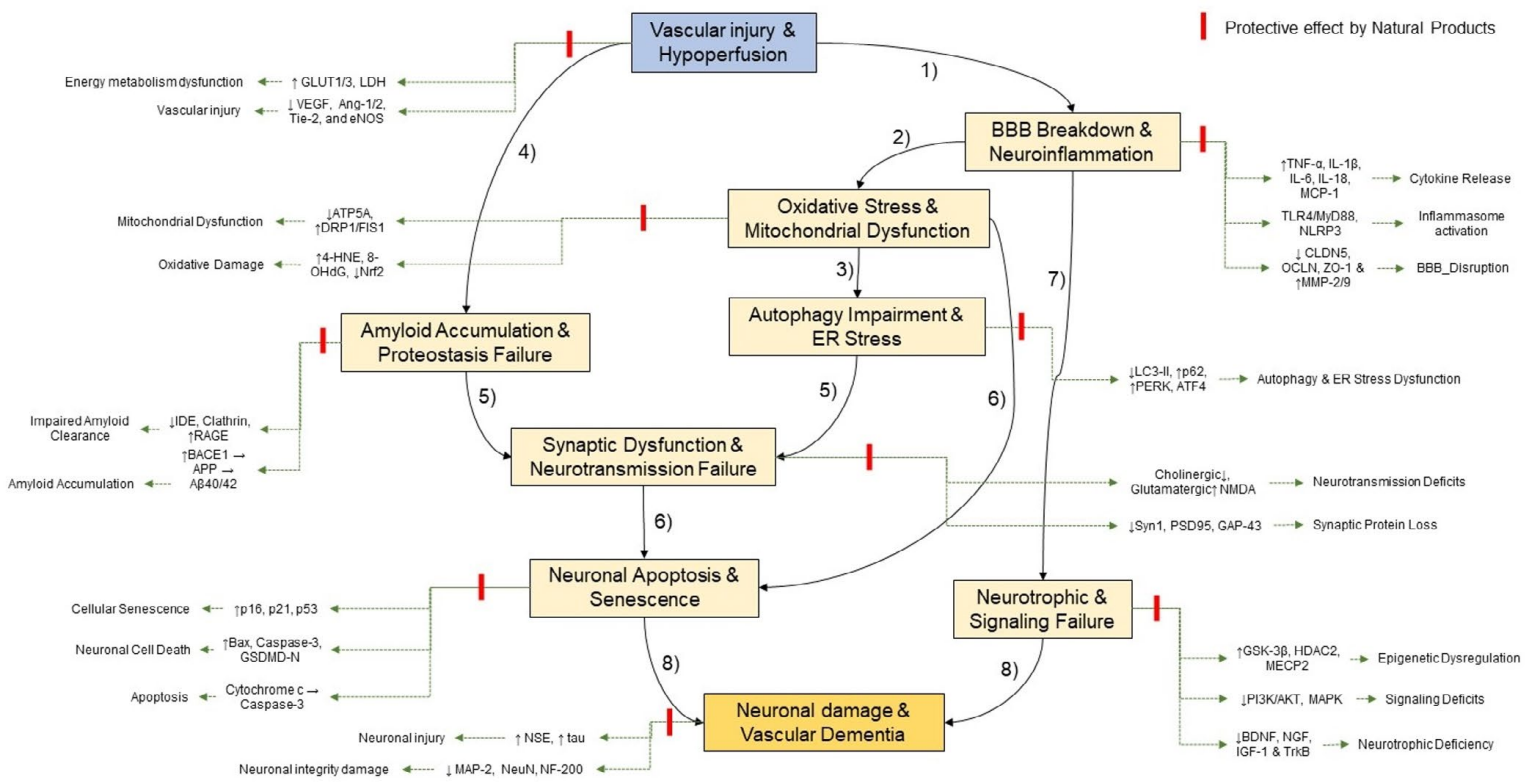
Key pathological pathways and molecular markers in VaD animal models and the protective effects of natural products. The numbers on the arrows indicate the pathological flow of VaD, and the red vertical line indicates the inhibitory effect of natural products (herbal formulas, single herbs, and phytochemicals).

## 4 PPI Network Analysis

PPI analysis revealed that Akt1 plays a central role in all studies utilizing herbal formulas, single herbs, and phytochemicals. In studies using herbal formulas, IL‐6 and caspase‐3 emerged as key proteins (Figure 2A), while those on single herbs and phytochemicals highlighted caspase‐3 and IL‐1β (Figure 2B,C). These findings suggest that the studies analyzed in this review primarily focused on proteins involved in cell survival, proliferation, apoptosis, and inflammatory responses. Furthermore, our bioinformatic analysis provides a hypothesis‐generating summary of the targets reported in those studies. Prominent nodes identified in the network, such as Akt1, IL‐1β, IL‐6, and caspase‐3, represent major nodes of interest that have been preferentially selected for measurement in previous studies, reflecting the aggregated focus of existing experimental studies rather than providing independent evidence of mechanistic centrality (Figure 2D). These proteins are closely interconnected and play critical roles in balancing cell survival, apoptosis, and inflammatory responses. Akt1 functions as a cell survival regulator, while caspase‐3 induces programmed cell death (apoptosis), illustrating their opposing roles in cellular homeostasis. IL‐1β and IL‐6 act as proinflammatory cytokines, actively promoting immune responses and inflammation. Conversely, Akt1 exerts anti‐inflammatory effects by mitigating excessive inflammatory signaling. Additionally, IL‐1β and IL‐6 induction can influence caspase‐3 activation, and heightened inflammatory responses may result in an increased apoptotic rate. This intricate regulatory network underscores the importance of coordinated protein modulation in VaD pathogenesis. Understanding these interactions provides valuable insights into potential therapeutic strategies and drug development that target neuroinflammation and cellular homeostasis in VaD.

**FIGURE 2:**
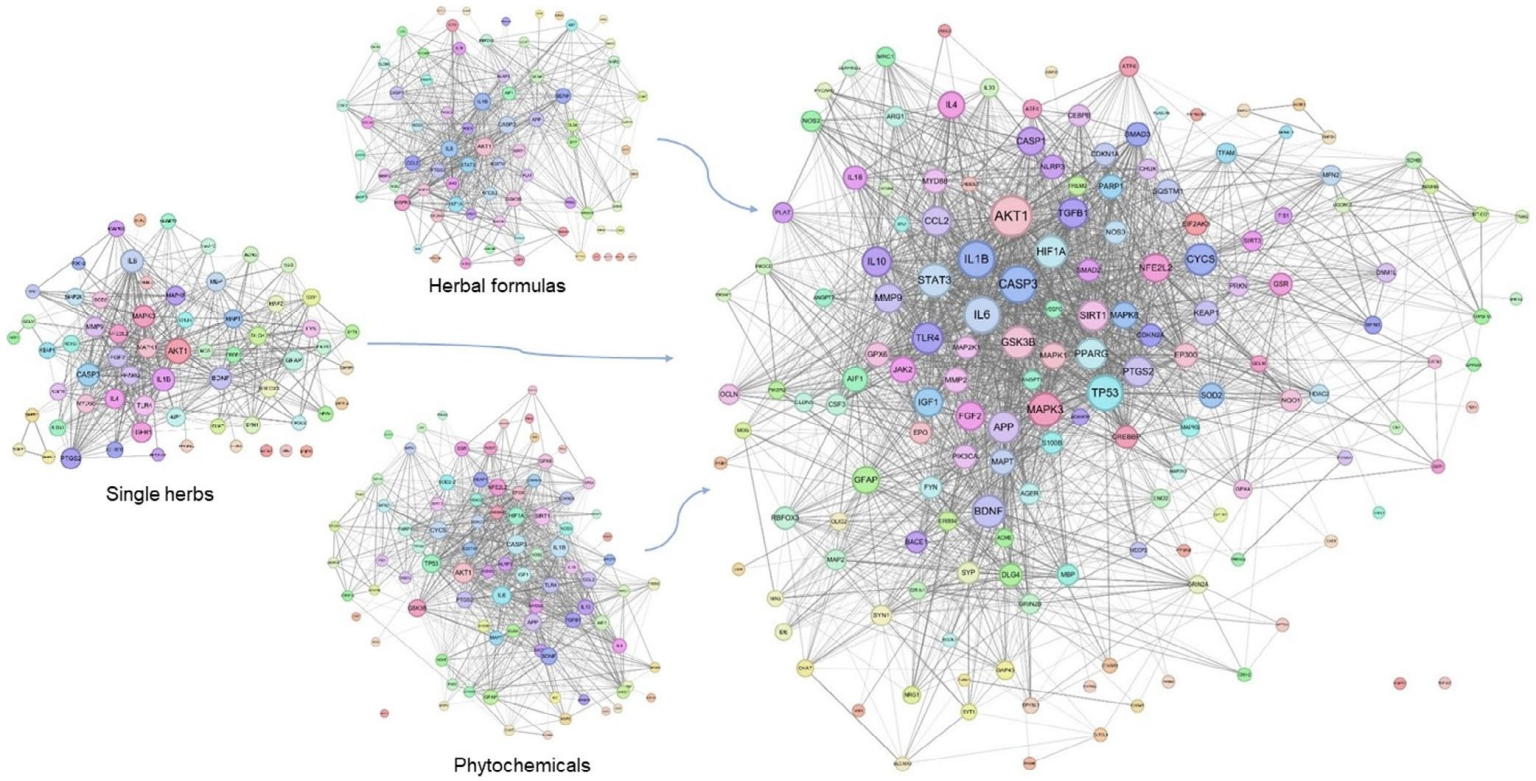
Multi‐layer PPI networks for herbal formulas (A), single herbs (B), phytochemicals (C), and the fully integrated network (D). In each panel, nodes represent protein targets and edges indicate predicted PPI; node size is scaled to degree (number of connections). These networks demonstrate the multi‐target landscape of natural products, highlighting how diverse herbal interventions converge on common survival and inflammatory hubs. The integrated network (D) provides a conceptual framework illustrating that natural products do not act on isolated pathways but rather modulate a cohesive inter‐protein interactome crucial for neuroprotection in VaD. (A) The herbal formula‐level network encompasses a broad, highly interconnected module in which survival‐ and inflammation‐related kinases (Akt1, MAPK1), pro‐inflammatory cytokines (IL‐1β, IL‐6, TNF‐α), and apoptosis regulators (caspase‐3, p53) form central hubs, reflecting the formulas' multitarget engagement of growth, death, and immune pathways. (B) The single herb‐level network retains Akt1, caspase‐3, and IL‐1β as key nodes but shows fewer peripheral connections, highlighting each herb's focus on core survival/apoptosis and stress/response circuits (e.g., oxidative stress and immune effector functions). (C) The phytochemical‐level network is the sparsest, with caspase‐3, IL‐6, and TNF‐α as the dominant interaction points; this indicates that purified phytochemicals exert highly targeted effects on apoptosis and cytokine signaling. (D) The integrated network merges A–C to reveal a unified topology in which Akt1, caspase‐3, IL‐1β, IL‐6, TNF‐α, and p53 emerge as the most connected hubs across all layers. This consolidation of targets mirrors our GO/KEGG enrichment findings—centering on oxidative‐stress defense, apoptosis modulation, and neuroinflammation pathways—and nominates these six proteins as prime mediators in the herbal ingredients with neuroprotective efficacy.

As shown in Figure 3, GO enrichment of herbal formulas, single herbs, and phytochemicals converges on the pathways that regulate cell survival, apoptosis, and inflammation—yet each dataset displays its own characteristics. In the herbal formula dataset (Figure 3A), GO enrichment highlights a broad system‐level impact on ‘survival–death–inflammation’ networks. In terms of Biological Process (BP), “cell proliferation,” “apoptotic process,” and “inflammatory response” emerge as the top hits, reflecting simultaneous modulation of growth, programmed cell death, and immune activation. Molecular Function (MF) enrichment is dominated by “protein kinase activity” and “cytokine activity,” underscoring the herbal formulas' ability to both engage key signaling kinases (e.g., Akt1) and tune proinflammatory mediators. Cellular Component (CC) terms split between the cytosol and extracellular region, indicating that the herbal formulas perturb both intracellular signaling hubs and secreted cytokines. When mapped onto KEGG pathways, the herbal formulas show particularly strong enrichment of PI3K‐Akt, TNF, and HIF‐1 signaling axes, mirroring the GO findings and pointing to coordinated regulation of redox balance, survival signaling, and neuroinflammatory circuits. These bioinformatic outputs largely corroborate well‐established pathological routes in vascular pathology, specifically the PI3K‐Akt, TNF, and oxidative stress‐related signaling pathways. However, the conceptual importance of our analysis lies in illustrating how natural products interface with the complex interconnection of these cascades specifically within the context of CCH models. Rather than acting on isolated targets, the results suggest that these interventions exert their protective effects by modulating the cross‐talk between survival‐promoting signaling (Akt1) and pro‐inflammatory cascades (TNF/NF‐κB), providing a structural overview of the multi‐target landscape in current herbal research.

**FIGURE 3:**
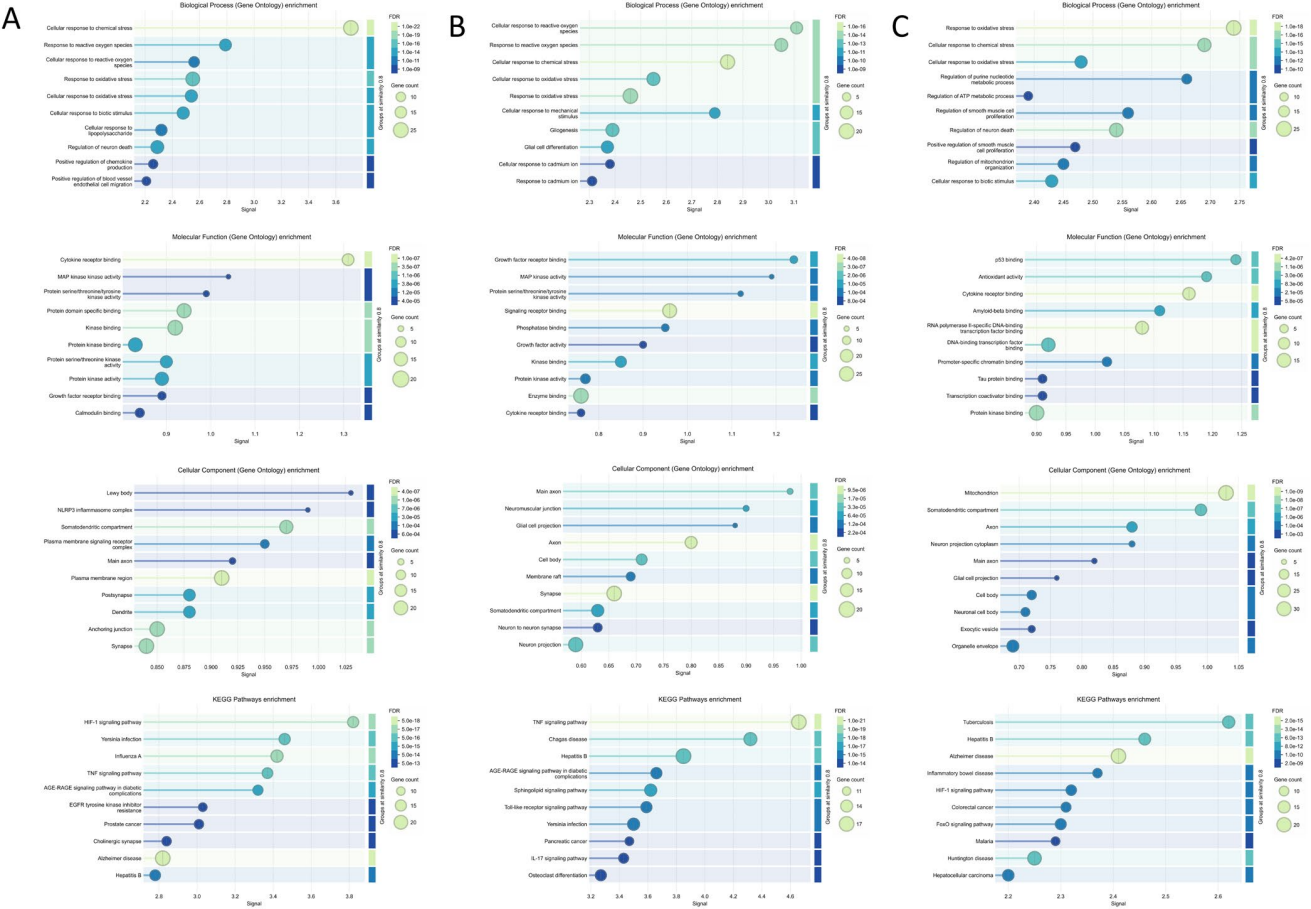
Integrated GO and KEGG enrichment of proteins regulated by herbal formulas (A), single herbs (B), and phytochemicals (C). Each panel contains four bubble‐chart subplots for GO Biological Process (BP), Molecular Function (MF), Cellular Component (CC), and KEGG pathway terms. In every chart, the x‐axis denotes enrichment significance (−log_10_ FDR), bubble size corresponds to the number of proteins annotated to each term, and color intensity reflects the FDR value. These enrichment analyses largely corroborate well‐established pathological routes in vascular dementia, such as the PI3K–Akt, TNF, and oxidative stress‐related pathways. Rather than revealing entirely novel mechanisms, these figures serve as a hypothesis‐generating summary that underscores the convergence of natural product research on core neuroinflammatory and apoptotic cascades in chronic cerebral hypoperfusion models. FDR, false discovery rate. (A) Herbal formulas drive broad BP enrichment in chemical/oxidative‐stress responses, apoptosis and inflammation; MF hits include cytokine receptor and protein kinase activities; CC terms span Lewy bodies, the NLRP3 inflammasome, neuronal projections, and plasma‐membrane complexes; KEGG pathways highlight HIF‐1, TNF, AGE–RAGE, Alzheimer's disease, and viral‐response modules. (B) Single herbs retain apoptosis and inflammation signals while increasing cellular response to oxidative stress and immune effector processes; MF emphasizes kinase‐ and cytokine‐receptor binding; CC shifts toward mitochondrial membrane and neuronal compartments; KEGG enrichment includes TNF, IL‐17, Toll‐like receptor, AGE–RAGE, and hypoxia signaling pathways. (C) Phytochemicals yield a narrow profile dominated by apoptotic process and cytokine‐mediated signaling (BP), caspase activation and receptor binding (MF), extracellular region localization (CC), and disease‐specific KEGG maps (e.g., hepatitis B, tuberculosis, Huntington's disease) together with HIF‐1 signaling.

In the single herb dataset (Figure 3B), at the BP level, the strongest enrichments involve cellular responses to chemical and oxidative stress, “cellular response to reactive oxygen species,” “cellular response to oxidative stress,” and “response to oxidative stress,” alongside developmental and remodeling processes such as gliogenesis and glial‐cell differentiation. MF terms peak in signal‐transducer interactions, notably “growth factor receptor binding,” “MAP kinase activity,” “protein serine/threonine/tyrosine kinase activity,” “kinase binding,” and “cytokine receptor binding,” underscoring the direct engagement of survival‐ and inflammation‐related kinases and receptors. CC hits localize predominantly to neuronal microenvironments—main axon, neuromuscular junction, glial projections, synapse, somatodendritic compartment, and membrane rafts—highlighting the targeted modulation of neuron–glia interfaces and membrane signaling platforms. KEGG pathway mapping mirrors these GO themes: inflammatory and stress‐response routes, such as TNF signaling, IL‐17 signaling, TLR signaling, and sphingolipid signaling, are among the top hits, as are oxidative/inflammatory axes like the AGE‐RAGE pathway in diabetic complications. Disease‐specific modules (Chagas disease, Hepatitis B, and pancreatic cancer) also emerge, reflecting shared inflammatory mediators. Collectively, this integrated GO/KEGG profile reveals that single‐herb interventions and their bioactive constituents act in concert to bolster antioxidant defenses, fine‐tune kinase‐driven survival and apoptosis circuits, remodel neuroglial connectivity, and temper proinflammatory networks, thus providing a mechanistic basis for their observed efficacy in VaD.

Figure 3 C presents the integrated enrichment across GO and KEGG. In the BP panel, the strongest enrichments center on redox homeostasis and survival: “cellular response to oxidative stress,” “cellular response to chemical stress,” “response to reactive oxygen species,” “regulation of purine nucleotide metabolic process,” “regulation of ATP metabolic process,” “regulation of neuron death,” “positive regulation of smooth muscle cell proliferation,” and “regulation of mitochondrion organization.” These terms underscore the coordinated control of oxidative‐stress defenses, energy metabolism, and cell‐survival/proliferation circuits. At the MF level, enriched terms include “p53 binding,” “antioxidant activity,” “cytokine receptor binding,” “amyloid‐β binding,” “DNA‐binding transcription factor binding,” “promoter‐specific chromatin binding,” “tau protein binding,” “transcription coactivator binding,” and “protein kinase binding.” This profile highlights direct engagement with stress‐response factors, immune receptors, neurodegeneration markers, and key signaling kinases. CC enrichment localizes predominantly to neuronal microenvironments and trafficking sites: “mitochondrion,” “somatodendritic compartment,” “axon,” “neuron projection cytoplasm,” “main axon,” “glial cell projection,” “cell body,” “exocytic vesicle,” and “organelle envelope.” These hits point to both subcellular organelles that manage oxidative stress and neuronal projections where synaptic and vesicular signaling occur. Finally, KEGG pathway analysis identifies significant associations with infectious and neurodegenerative disease modules (such as tuberculosis, hepatitis B, Alzheimer's disease, inflammatory bowel disease, colorectal cancer, malaria, Huntington disease, and hepatocellular carcinoma) alongside core stress/survival pathways—HIF‐1 and FoxO signaling. Collectively, this integrated enrichment landscape reveals that complex herbal formulas and their phytochemicals converge on oxidative‐stress defense, metabolic regulation, neuroprotection, and immune modulation, providing a unified mechanistic framework for their efficacy in VaD.

In the fully integrated analysis combining all three datasets—herbal formulas, single herbs, and phytochemicals—GO enrichment converges on a tight neuroprotective signature (Figures 2 and S1). At the BP level, the top hits are dominated by oxidative‐stress and survival/remodeling themes (“cellular response to oxidative stress,” “response to reactive oxygen species,” “regulation of neuron death,” and “positive regulation of endocytosis”), reflecting a coordinated effort to restore redox balance, prevent neurodegeneration, and support membrane trafficking. MF enrichment centers on protein–protein and receptor interactions—“kinase binding,” “protein kinase binding,” “growth factor receptor binding”, and “cytokine receptor binding”—underscoring the direct modulation of key survival kinases (e.g., Akt1) and inflammatory receptors (e.g., IL‐1R/IL‐6R). CC terms localize predominantly to neuronal microdomains (axon, post‐synapse, and somatodendritic compartment) and the extracellular region, highlighting that both synaptic/axon‐based signaling hubs and secreted mediators are targeted by the treatments. When these GO findings are projected onto KEGG pathways, stress and inflammation circuits such as HIF‐1 and FoxO signaling, the AGE‐RAGE axis in diabetic complications, Alzheimer's disease modules, and TNF signaling emerge as the most significant routes. Collectively, this integrated GO/KEGG profile shows that multi‐component herbal formulas—and ultimately their herbal ingredients and phytochemicals—synergistically bolster antioxidant defenses, fine‐tune apoptotic thresholds (via caspase‐3), and temper neuroinflammation, all via an Akt1‐centered network. These mechanistic insights offer a unified molecular framework for the neuroprotective efficacy observed in our VaD model and nominate Akt1, caspase‐3, IL‐1β, and IL‐6 as prime targets for future intervention.

It is important to acknowledge the intrinsic circularity of this bioinformatic approach; since the PPI and enrichment analyses are based on targets previously selected by primary investigators, the results inherently reflect existing research trends. Therefore, the central hubs identified in our networks should be interpreted as consolidated summaries of what has been most frequently studied in the field of natural products for VaD, serving as a guide for future hypothesis generation rather than definitive causal drivers.

## 5 Feasible Animal Modeling to Induce CCH for an In Vivo VaD Experiment

In this review, the two most widely used preclinical paradigms for modeling VaD—BCAS and BCCAo (2VO)—were deliberately emphasized, as they encompass the spectrum of CCH syndromes most relevant to human subcortical ischemic VaD (Venkat et al. 2015; Tiwari et al. 2021; Hattori et al. 2016).

BCCAo in rats produces an abrupt and near‐complete (> 80%) reduction of forebrain perfusion that remains stable for weeks, thereby modeling global oligemia (Wakita et al. 1995). This persistent hypoperfusion elicits robust astrocyte and microglial activation, BBB disruption, and widespread white‐matter vacuolation by 7–28 days post‐occlusion (Wakita et al. 1995; Ueno et al. 2002). In contrast, BCAS in mice induces a more moderate (~30%–40%) drop in cerebral blood flow within hours, followed by partial recovery to ~80% of baseline over four weeks (Shibata et al. 2004). Although this biphasic hemodynamic course complicates the distinction between acute ischemic injury and chronic hypoperfusion effects, BCAS yields selective white matter rarefaction and working memory deficits that closely mirror the pattern of subcortical ischemic vascular cognitive impairment (Nishio et al. 2010; Shibata et al. 2007).

Both models produce learning and memory impairments, but their temporal trajectories and task sensitivities differ. BCCAo rats typically exhibit spatial memory deficits in the Morris water maze and radial arm maze by one week post‐occlusion, whereas BCAS mice show robust working memory (Y‐maze) and motor (rotarod) deficits by 28 days, with reference memory impairment emerging only after several months (Nishio et al. 2010; Shibata et al. 2007). This divergence reflects the underlying histopathology: BCCAo induces more widespread neuronal loss, including hippocampal CA1 damage, whereas BCAS primarily injures deep white matter, preserving hippocampal integrity in the early weeks. Researchers should therefore align model choice with the cognitive domains of interest: BCAS is better for subcortical executive function studies, whereas BCCAo is better for global learning and memory investigations.

BCAS mice have a perioperative mortality rate of ~10%–20%, depending on microcoil diameter, yet yield highly consistent stenosis and are readily applied to transgenic strains. BCCAo in rats has a minimal mortality rate but presents inter‐animal variability in infarct volume and location, necessitating strict lesion size inclusion criteria and potentially confounding visual‐pathway function (Wakita et al. 1995; Shibata et al. 2004). The BCAS model's compatibility with genetically manipulated mice makes it the preferred choice for mechanistic explorations of specific molecular pathways, whereas BCCAo remains valuable for evaluating candidates under conditions of severe global oligemia.

Overall, these findings support a two‐stage preclinical workflow: (1) initiating high‐throughput screening in BCAS mice to evaluate anti‐inflammatory, myelin‐protective, or cerebrovascular‐targeted compounds under a controlled stenotic condition and (2) subsequently validating promising candidates in BCCAo rats to confirm efficacy under more severe oligemia and infarct heterogeneity. This approach leverages the reproducibility and genetic tractability of BCAS with the global ischemic context and robust histological lesions of BCCAo, thereby strengthening the predictive validity of preclinical results. Tables S5 and S6 summarize the major rodent models and the two most frequently used models for inducing vascular cognitive impairment and dementia, respectively.

## 6 Interconnected Net‐Work Among VaD‐Protective Natural Products

Figure 4 and Table S7 illustrate the complex interconnected network between various herbal formulas and their specific ingredients. By mapping out the network analysis results, herbal ingredients were categorized into four distinct clusters (G1–G4) based on their relevance to herbal formulas. The ingredients in Group 1 (G1) serve as a central hub and correspond closely to the Tongqiao Huoxue Decoction and Danggui Shaoyao San. Within this group, Angelicae Sinensis Radix and Chuanxiong Rhizoma are linked to their active phytochemicals, ligustilide and ligustrazine, respectively. Additionally, paeoniflorin is linked to Paeoniae Radix in G1, while gastrodin is associated with Gastrodiae Rhizoma in G4 (rather than Group 3 as previously stated). The Zishen Huoxue Decoction aligns with the ingredients in Group 2 (G2). This cluster prominently features Salviae Miltiorrhizae Radix et Rhizoma, which contains active phytochemicals such as tanshinol, tanshinone IIA, and salvianolic acid B. Other anti‐VaD phytochemicals associated with Group 2 include betaine and schisandrin B. Group 3 (G3) exactly matches the composition of the Tongqiao Yizhi Granule, consisting of five herbal ingredients: Astragali Radix, Puerariae Lobatae Radix, Hirudo, Pheretima, and Ganoderma. In this group, puerarin acts as a key anti‐VaD phytochemical. The ingredients in Group 4 (G4) most closely match the herbal composition of Choto San. Ginseng Radix, which contains the active ginsenosides Rg1 and Rd., is a central herb linked to these formulas that exhibit protective effects in VaD models. Furthermore, glycyrrhizic acid (glycyrrhizin) is identified as an active phytochemical linked to Glycyrrhizae Radix et Rhizoma located within the G1/G4 network intersection.

**FIGURE 4:**
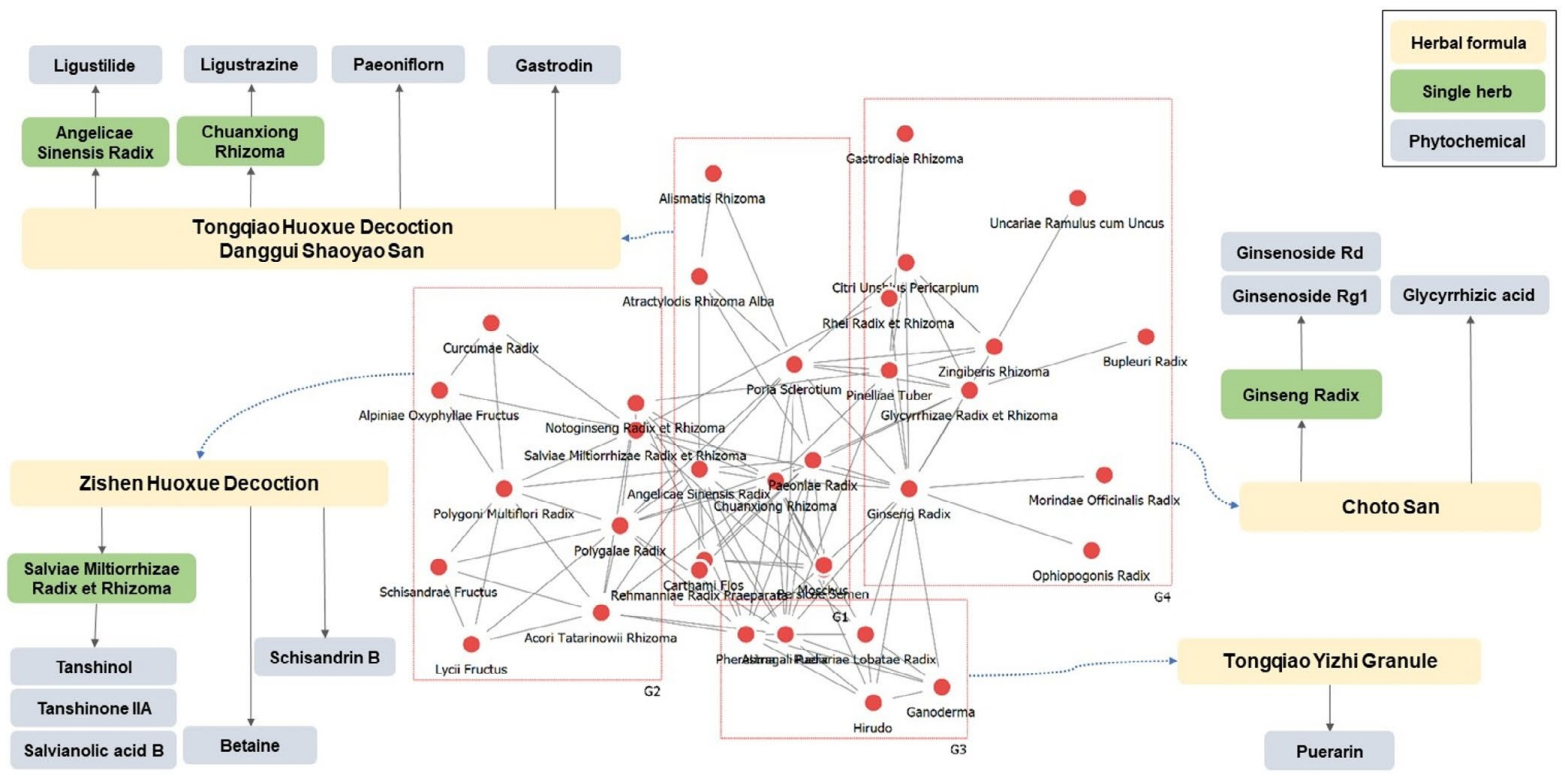
Network analysis‐based grouping of the herbal ingredients in the herbal formulas and matching each group to relevant herbal formulas with the single herbs and phytochemicals reported in the literature.

Through the network‐based categorization, we could observe core herbal formulas and their herbal ingredients exerting anti‐VaD effects. This observation provides insight for further application of those resources having various CNS‐protective mechanisms in various pathological animal models to anti‐VaD effects, such as the neuroprotective effects of Tongqiao Huoxue Decoction and Chuanxiong Rhizoma in cerebral ischemia rats (Li et al. 2017; Wang, Yao, et al. 2020), Choto San in mice with Aβ_1–42_‐induced cognitive impairment (Chen et al. 2016), Salviae Miltiorrhizae Radix et Rhizoma exerting anti‐coagulant effects via phospholipase C/protein kinase C signaling pathways (Fei et al. 2017). Meanwhile, single herbs included in the above groups but lacking reported anti‐VaD effects may serve as primary candidates for future investigation in VaD animal models. For example, Paeoniae Radix ranking third in usage frequency among the herbal formulas (Table S8) significantly suppressed hemorrhage‐induced neurodegeneration and caspase‐3 expression in the hippocampus and cerebral cortex of rats (Kim et al. 2004). Astragali Radix, ranked fourth in frequency, inhibits Aβ production, tau hyperphosphorylation, neuronal damage, neuroinflammation, and apoptosis in Alzheimer's disease animal models (Dong et al. 2022). Moreover, single herbs that serve as botanical sources of anti‐VaD phytochemicals warrant further investigation. Puerariae Lobatae Radix, a botanical source of puerarin, diminished oligomeric Aβ_25‐35_‐induced damages in Alzheimer's disease mice (Huang et al. 2019). Gastrodiae Rhizoma, a source of gastrodin, promoted AKT signaling‐based neuronal development in mice with D‐galactose‐induced memory damage (Hsu et al. 2021). Finally, other classes of phytochemicals derived from the aforementioned single herbs may effectively contribute to protective signaling pathways in VaD models. Paeonol, a phenolic compound in Paeoniae Radix, suppressed Aβ deposition, neural loss, microglia activation, and neuroinflammation in the APP/PS1 mouse brain (Meng et al. 2021). Ginseng Radix‐derived triterpenoids exerted neuroprotective effects in rats with middle cerebral artery occlusion‐induced cerebral ischemic injury (Cheng et al. 2019). This interconnected network‐based approach for grouping by herbal ingredients and finding core herbal formulas can expand the feasibility of natural products in investigating the protective mechanisms in VaD animal models. However, it should be clarified that the relevance between network groups and core herbal formulas is exploratory and based on co‐occurrence patterns only, and does not imply full pharmacological equivalence or identical clinical indications.

Several studies provide the safety information of aforementioned natural products through toxicological assessment. Repeated oral administration of Angelicae Sinensis Radix up to 2000 mg/kg/day for 4 weeks showed no toxicological features in rats (Lim and Kim 2014). Intravenous injection of Salviae Miltiorrhizae Radix et Rhizoma exhibited no‐observed‐adverse‐effect‐level at 5.76 g/kg/day for 13 weeks to rats (Wang et al. 2012). Intraperitoneal injection of Astragali Radix showed a safe dosage range at 5.7–39.9 g/kg/day for rats and 2.85–19.95 g/kg/day for Beagle dogs for 3 months without toxic features (Yu et al. 2007). Oral dosages of 20 (S)‐ginsenoside Rg3 (up to 20 mg/kg) and ginsenoside Re (up to 375 mg/kg/day) had non‐toxic effects for 26 weeks to Beagle dogs and rats, respectively (Gao et al. 2020; Lu et al. 2012).

## 7 Conclusion and Perspectives

Natural products demonstrated neuroprotective effect against VaD animal models by enhancing vascular regeneration and BBB stability, and reducing neuroinflammation, oxidative stress, and mitochondrial dysfunction. These effects enhanced synaptic plasticity, autophagy, proteostasis, and neurotrophic signaling, and inhibited neuronal apoptosis. By promoting overall neuronal survival, natural products improved cognitive deficits in VaD animal models.

Among the proteins in molecular signaling pathways, Akt1 was highlighted as a central regulator in VaD models, coordinating cell survival, apoptosis, and inflammatory responses alongside other key proteins, such as caspase‐3, IL‐1β, and IL‐6. GO and pathway enrichment revealed that multi‐ingredient herbal formulas and their active phytochemicals converged on neuroprotective mechanisms involving oxidative stress defense, kinase signaling, and immune modulation. These findings provide a unified molecular framework for anti‐VaD effects and suggest that targeting the Akt1‐centered network and its association with inflammatory and apoptotic pathways could guide future drug development strategies.

The two principal rodent models for VaD, BCAS and BCCAo, closely resemble clinical conditions by distinctly mimicking key aspects of CCH. BCAS is ideal for mechanistic studies due to its reproducibility and compatibility with transgenic mice, whereas BCCAo is applicable for studying severe global ischemia. A two‐stage preclinical strategy, initial screening in BCAS followed by validation in BCCAo, can offer a robust and translational workflow for investigating effective mechanisms against VaD models.

In addition, established and underexplored herbs, particularly those with known efficacy in related neurological disorders, such as ischemic brain injury or Alzheimer's disease, may hold promising mechanistic potential for VaD. Individual phytochemicals and their signaling mechanisms (e.g., AKT, TLR4/MyD88, and SIRT1) present targets for future mechanistic approaches.

Despite overall investigation of protective mechanisms of natural products against VaD animal models, this study has a few limitations: (1) female animal models were absent in all the articles analyzed except for one paper (Li, Ye, et al. 2024). This might lead to sex‐biased interpretation of results, which does not consider the pathophysiological differences such as hormonal influence, immunological and inflammatory varieties, or structural complexity; (2) the majority (≈88.8%) of animal species used were rats (SD rats, 65.3%; Wistar rats, 23.5%) followed by mice (C57BL/6 mice, 8.2%; ICR mice, 2.0%) and gerbils (1.0%). Such an unequal distribution among the animal species, especially SD rat‐focused models, can narrow the application of those mechanistic investigations to other species or strains having the vast genetic variations and diverse biological pathways; (3) the administration routes of natural products reported intragastric (80.5%), intraperitoneal (17.2%), and intravenous administration (2.3%). The intragastric administration has been selected predominantly as it mimics oral intake and is easier for long‐term dosing regimens. However, it also enables the natural product to pass through the entire digestive system and subsequently the metabolism before it reaches the bloodstream or the brain. This sometimes makes it difficult to find out contributing factors for the protective effect of natural products; (4) the Morris water maze test has been regarded as the “gold standard” for highly reliable and validated behavioral tests of spatial learning and memory in neurodegenerative rodent models. However, exclusive reliance on a single behavioural test might fail to capture diverse aspects of cognitive functions, such as recognition and working memories.

Taken together, these findings highlight the potential of natural products to treat VaD via multiple pathological mechanisms, thereby necessitating further investigation of their neuroprotective properties. Therefore, future research should focus on exploring underreported herbs and their bioactive phytochemicals to expand therapeutic options and clarify the molecular pathways involved in VaD treatment.

## Author Contributions

**Jung‐Hoon Kim:** conceptualization, methodology, data curation, visualization, investigation, writing – original draft, writing – review and editing, supervision. **Suin Cho:** conceptualization, methodology, investigation, visualization, writing – original draft, writing – review and editing, supervision, data curation, software. **Yongtaek Oh:** methodology, writing – original draft, visualization, software.

## Funding

The authors have nothing to report.

## Ethics Statement

The authors have nothing to report.

## Consent

The authors have nothing to report.

## Conflicts of Interest

The authors declare no conflicts of interest.

## Supporting information


**Figure S1:** Integrated GO and KEGG enrichment across proteins regulated by herbal formulas, single herbs and phytochemicals. (A–C) Bubble charts of GO BP, MF CC terms; (D) KEGG pathway enrichment; (E) schematic network integrating HIF‐1, a key GO terms and pathways. On each chart, the x‐axis denotes enrichment significance (−log_10_ FDR), bubble size represents the number of proteins annotated to that term, and color intensity reflects FDR. BP highlights oxidative‐stress responses, neuron survival and endocytosis; MF centers on kinase/receptor binding; CC localizes to neuronal projections and extracellular milieu; KEGG emphasizes HIF‐1, FoxO, AGE‐RAGE, Alzheimer's and TNF signaling.
**Table S1:** Target proteins structured in the integrated mapping.
**Table S2:** Pharmacological effects of herbal formulas in animal models of VaD (ordered by publication year).
**Table S3:** Pharmacological effects of single herbal medicines in animal model of VaD (ordered by publication year).
**Table S4:** Pharmacological effects of phytochemicals in animal model of VaD (ordered by publication year).
**Table S5:** Overview of common rodent models of VaD.
**Table S6:** Summary of the two most frequently used rodent models of VaD.
**Table S7:** Network group of herbal ingredients.
**Table S8:** Usage frequency (≥ 5) of herbal ingredients occurring in the herbal formulas.

## Data Availability

The data that support the findings of this study are available from the corresponding author upon reasonable request.
