# Supplementary material for: Protective Potentials of Natural Products Against Vascular Dementia: A Comprehensive Review of Experimental Studies
Source: Phytother Res. 2026 Sep 13;40(11):6535–52. doi: 10.1002/ptr.70448 (PMC13753685; doi:10.1002/ptr.70448)
Supplement: Supplementary file 1 [file PTR-40-6535-s001.docx]

**Supporting Information**

Protective Potentials of Natural Products against Vascular Dementia: A Comprehensive Review of Experimental Studies

Jung-Hoon Kim^1^, Yongtaek Oh^2^, Suin Cho^1^

^1^Division of Pharmacology, School of Korean Medicine, Pusan National University, Yangsan 50612, Republic of Korea

^2^Department of Diagnostics, College of Korean Medicine, Woosuk University, Jeonju, 55338, Republic of Korea

Correspondences: Jung-Hoon Kim (kmsct@pusan.ac.kr) and Suin Cho (sicho@pusan.ac.kr)

**Figure S1**. Integrated GO and KEGG enrichment across proteins regulated by herbal formulas, single herbs and phytochemicals.

(A–C) Bubble charts of GO BP, MF CC terms; (D) KEGG pathway enrichment; (E) schematic network integrating HIF-1, a key GO terms and pathways. On each chart, the x-axis denotes enrichment significance (–log₁₀ FDR), bubble size represents the number of proteins annotated to that term, and color intensity reflects FDR.

BP highlights oxidative‐stress responses, neuron survival and endocytosis; MF centers on kinase/receptor binding; CC localizes to neuronal projections and extracellular milieu; KEGG emphasizes HIF-1, FoxO, AGE-RAGE, Alzheimer’s and TNF signaling.

**Table S1**. Target proteins structured in the integrated mapping

| **Natural product** | **Target protein** |
| --- | --- |
| Herbal formula | Aβ_42_, AKT1, Ang-1, Ang-2, APP, ASC, ATF4, ATF6, Bax, Bcl-2, BDNF, Beclin1, CaMKII, cAMP, Caspase-1, Caspase-3, CAT, ChAT, Clathrin, Claudin-5, COX-2, CREB, elF2a, eNOS, EPO, ERK, GAP-43, GluA2, GluN1, GluN2B, GPx-1, GRP78 (Bip), GSDMD-N, HIF-1α, HO-1, Iba-1, IkBα, IL-18, IL-1β, IL-6, iNOS, JAK2, JNK, LC3-2/1, MAP-2, MBP, M-CSF, M-CSF receptor, miR-124-3p, MMP, MMP-2, Muscarinic m3, Muscarinic m5, NF-kB, NLRP3, NMDAR 1, NMDAR 2A, NMDAR 2B, NMDAR1, NQO1, Nrf2, Occluding, p38, p56, p62, p65, PAI-1, p-AKT, Parkin, p-CREB, p-CRMP2, p-EPOR, p-ERK 1/2, p-GSK-3β, PIK3Cα, PINK1, p-IRE1α, p-JNK, PKAc, p-MEK 1/2, p-p38 MAPK, p-PERK, PSD95, p-STAT5, RAB5B, SIRT1, STAT3, SYN, SYP, Tie-2, TNF, TNF-α, t-PA, TrkB, VEGF, ZO-1 |
| Single herb | AKT, Ang-2, Arginase-1, Bax, Bcl-2, BDNF, bFGF, C/EBPβ, CaMKII-α, caspase-3, CD68, ChAT, COX-2, DPYSL2, GAD65, GLS, GSH, GSK3β ser9, HO-1, Iba-1, IkB-α, IL-1β, IL-4, IL-6, iNOS, Keap1, Lingo-1, MAG, MAP-2, MAPKK 1, MBP, MMP9, MOG, MyD88, NF-200, NF-kB, NGF, NMDA2B, Nrf2, NRG-1, p110α, p25, p35, p65, p85, p-AKT, p-CREB, p-ErbB4, p-ERK, p-ERK1/2, p-Fyn, p-GSK3β, PI3K, p-JNK, p-JNK1/2, , PP2A-sub B, p-p38, p-p38 MAPK, PPAR-γ, PSD-95, p-tau, RAGE, ROCK2, Sgms1, SMPD1, SMPD2, SOD, SYN, Synapsin 1, Synaptophysin, Synaptotagmin-1, TGF-β, TLR4, TNF-α, TrkB, TRLR4, TXNL 1, VEGF, Wnt3 |
| Phytochemical | 4-HNE, Aβ_1-40_, Aβ_1-40/42_, Aβ_1-42_, AChE, AcSOD2, ADAM10, APP, Arg-1, ASC, BACE-1, Bax, Bcl-2, BDNF, Beclin-1, C3d, Calpastatin, Caspase-1, Caspase-3, CBP, CD206, ChAT, COX-2, Cyto-c, DRP1, Fis1, γ-GCS, GFAP, GLUT1, GLUT3, GPR30, GPx4, GSDMD-N, Hcy, HDAC2, HIF-1α, HO-1, HUMMR, Iba1, IDE, IGF-1, IL-10, IL-18, IL-1β, IL-4, IL-6, iNOS, Keap1, LC3I, LC3II, MAP2, MBP, MCP-1, Mfn1/2, MyD88, NF-kB, NLRP3, NMDAR2A, NMDAR2B, NQO-1, NR2A, NR2B, NR2B-pTyr1472, Nrf2, NSE, p16, p21, P2X7R, p300, p50, p53, p62, p65, p-AKT, p-AMPK, PARP, p-CaMKII, p-CREB, p-ERK, p-GSK 3β, pMeCP2, p-NF-kB, p-Nrf2, p-p38, p-p38, MAPK, p-PI3K, PSD93, PSD-95, p-Smad2, p-Smad-3, p-STAT 3 Tyr705, p-STAT3 Ser727, p-tau, p-tau217, p-tau396, S100B, SIRT1, SIRT3, Spectrin aII, STAT3, SYN-1, TFAM, TGF-β1, TLR4, TNF-α, TRAK2, TREM2, TrkB, TRPM2, TUNEL, VAChT |

4-HNE, 4-hydroxy-2-nonenal; Aβ, amyloid beta; AChE, acetylcholinesterase; AcSOD2, acetylated superoxide dismutase 2; ADAM10, a disintegrin and metalloproteinase domain-containing protein 10; AKT, protein kinase B (PKB); Ang, angiopoietin; APP, amyloid precursor protein; Arg-1, arginase 1; ASC, apoptosis-associated speck-like protein containing a CARD; ATF, activating transcription factor; BACE1, β-site APP-cleaving enzyme 1; Bax, Bcl-2-associated X protein; Bcl-2, B-cell lymphoma-2; BDNF, brain-derived neurotrophic factor; bFGF, basic fibroblast growth factor; C/EBPβ, CCAAT/enhancer binding protein beta; C3d, complement component 3d; CaMKII-α, calcium/calmodulin-dependent protein kinase II alpha; CAT, catalase; CBP, CREB-binding protein; ChAT, choline acetyltransferase; COX-2, cyclooxygenase-2; CREB, cAMP response element-binding protein; Cyto-c, cytochrome c; DPYSL2, dihydropyrimidinase-like 2; DRP1, dynamin-related protein 1; EPO, erythropoietin; ERK, extracellular signal-regulated kinase; Fis1, mitochondrial fission 1 protein; GAD65, glutamate decarboxylase 65; GAP-43, growth-associated protein 43; GFAP, glial fibrillary acidic protein; GLS, glutaminase; GluA2, glutamate receptor AMPA 2, GluN1, glutamate receptor NMDA 1; GluN2B, glutamate receptor NMDA 2B; GLUT, glucose transporter; GPR30, G-protein coupled receptor 30; GPx, glutathione peroxidase; GRP78 (Bip), glucose-regulated protein 78 (binding immunoglobulin protein); GSDMD-N, gasdermin D, N-terminal fragment; GSH, glutathione; GSK3β ser9, glycogen synthase kinase 3 beta (Ser9); Hcy, homocysteine; HDAC2, histone deacetylase 2; HIF-1α, hypoxia-inducible factor 1-alpha; HO-1, heme oxygenase-1; HUMMR, huntingtin-associated protein 1; Iba1, ionized calcium-binding adapter molecule 1; IDE, insulin-degrading enzyme; IGF-1, insulin-like growth factor 1; IkBα, inhibitor of nuclear factor kappa-B alpha; IL, interleukin; iNOS, inducible nitric oxide synthase; JAK2, Janus kinase 2; JNK, c-Jun N-terminal kinase; Keap1, Kelch-like ECH-associated protein 1; LC3-2/1, microtubule-associated protein 1 light chain 3; LC3I, microtubule-associated protein 1 light chain 3-I; LC3II, microtubule-associated protein 1 light chain 3-II; Lingo-1, leucine-rich repeat and Ig domain-containing protein 1; MAG, myelin-associated glycoprotein; MAP2, microtubule-associated protein 2; MAPKK 1, mitogen-activated protein kinase 1; MBP, myelin basic protein; MCP-1, monocyte chemoattractant protein-1; M-CSF, macrophage colony-stimulating factor; Mfn1/2, mitofusin 1/2; MMP, matrix metalloproteinase; MOG, myelin oligodendrocyte glycoprotein; Muscarinic m3, muscarinic acetylcholine receptor M3; Muscarinic m5, muscarinic acetylcholine receptor M5; MyD88, myeloid differentiation primary response 88; NF-200, neurofilament 200; NF-kB, nuclear factor kappa-light-chain-enhancer of activated B cells; NLRP3, NLR family pyrin domain containing 3; NMDAR, N-methyl-D-aspartate receptor subunit; NQO1, NAD(P)H quinone dehydrogenase 1; NR, NMDA receptor subunit; Nrf2, nuclear factor erythroid 2-related factor 2; NRG-1, neuregulin-1; p110α, catalytic subunit of phosphoinositide 3-kinase; p16, cyclin-dependent kinase inhibitor 2A; p21, cyclin-dependent kinase inhibitor 1A; P2X7R, purinergic receptor P2X7; p300, histone acetyltransferase p300; p53, tumor protein p53; p56, protein 56; p62, sequestosome 1; p65, RelA (subunit of NF-kB); p85, regulatory subunit of phosphoinositide 3-kinase; PAI-1, plasminogen activator inhibitor-1; Parkin, E3 ubiquitin-protein ligase Parkin; PARP, Poly (ADP-ribose) polymerase; p-Fyn, phosphorylated Fyn kinase; PIK3Cα, phosphatidylinositol 3-kinase catalytic subunit alpha; PINK1, PTEN-induced kinase 1; PKAc, protein kinase A catalytic subunit; p-MeCP2, phosphorylated methyl-CpG-binding protein 2; PP2A-sub B, protein phosphatase 2A subunit B; p-p38, phosphorylated p38 mitogen-activated protein kinase; p-p38 MAPK, phosphorylated p38 mitogen-activated protein kinase; PPAR-γ, peroxisome proliferator-activated receptor gamma; p-PI3K, phosphorylated phosphoinositide 3-kinase; PSD-95, postsynaptic density protein 95; p-tau, phosphorylated tau protein; RAB5B, Ras-related protein Rab-5B; RAGE, receptor for advanced glycation end-products; ROCK2, Rho-associated protein kinase 2; Sgms1, Sphingomyelin synthase 1; SIRT, Sirtuin; SMPD, sphingomyelin phosphodiesterase; SOD, superoxide dismutase; STAT3, signal transducer and activator of transcription 3; SYN, synapsin; SYP, synaptophysin; TGF-β, transforming growth factor beta; Tie-2, tyrosine kinase with immunoglobulin-like and EGF-like domains 2; TLR4, Toll-like receptor 4; TNF-α, tumor necrosis factor alpha; t-PA, tissue plasminogen activator; TRAK2, trafficking kinesin protein 2; TREM2, triggering receptor expressed on myeloid cells 2; TrkB, tropomyosin receptor kinase B; TUNEL, terminal deoxynucleotidyl transferase dUTP nick end labeling; TXNL 1, thioredoxin-like protein 1; VAChT, vesicular acetylcholine transporter; VEGF, vascular endothelial growth factor; Wnt3, wingless-type MMTV integration site family member 3; ZO-1, Zonula occludens-1.

**Table S2.** Pharmacological effects of herbal formulas in animal models of VaD (ordered by publication year)

| **Material** | **Animal (age/sex)**  **·Model**  **·Administration (route)** | **Cognitive ability** | **Organ** | ***in vitro* / Blood** | **Ref.** |
| --- | --- | --- | --- | --- | --- |
| Tong Qiao Huo Xue Decoction | SD rat (-/-)  ·Bilateral vessel occlusion  ·3, 6, and 12 g/kg/d for 28 days (oral) | **Morris water maze test**  ·Escape latency ↓  ·Time in the target quadrant ↑  ·Cross in target quadrant platform ↑  **Y maze test**  ·Entries into the new foreign arm ↑ | **Hippocampus**  ·Neurons with pyknotic nuclei, necrosis of brain tissue and partial liquefaction reversed  ·Dendritic length, spine density and synaptic loss normalized  ·GAP-43, SYP, PSD-95 proteins ↑  ·cAMP, PKAc, p-CREB, BDNF proteins ↑ | **HT22 cell** (OGD/R-induced injury cell model)  ·cAMP, PKAc, P-CREB, BDNF mRNA ↑  (Tong-Qiao-Huo-Xue Decoction cerebrospinal fluid) | Li, Ye, et al. 2024 |
|  | SD rat (-/male and female evenly)  ·Bilateral carotid artery ligation  ·3.01, 6.02, and 12.04 g/kg for 31 days (intragastric) | **Morris water maze test**  ·Escape latency and traveled distance ↓ | **Hippocampus**  ·Phosphorylation of PKA, CREB, CaMKII proteins ↑  ·NMDAR 1, NMDAR 2A, NMDAR 2B mRNAs ↑ | - | Ge et al. 2015 |
| Tongqiao Yizhi Granule | SD rat (8 weeks/male)  ·Permanent occlusion of two-vessel occlusion  ·6.2, 12.4, and 24.8 g/kg/d for 30 days (oral) | **Morris water maze test**  ·Escape latency ↓  ·Cross in target quadrant platform ↑  ·Dwell time in original target quadrant ↑ | **Hippocampus**  ·Cell swelling, release of contents, chromatin fixation, intact cell membranes recovered  ·Neuronal pyroptosis ↓, positive neurons ↑  ·P65, NLRP3, caspase-1, anti-GSDMD-N proteins and mRNAs ↓ | **Blood**  ·IL-18 IL-1β ↓ | Li, Hao, et al. 2024 |
|  | SD rat (-/male)  ·Permanent blockage of common carotid arteries on both sides  ·4.84 and 9.68 g/kg/d for 30 days (-) | **Morris water maze test**  ·Escape latency ↓  ·Cross in target quadrant platform ↑  ·Swimming time in quadrant III ↑ | **Hippocampus**  ·Dendritic spine density ↑  ·Synaptic ultrastructure recovered  ·GluA2, GluN1, GluN2B proteins ↑  ·GAP-43, SYP, PSD-95 proteins ↑  ·p-MEK1/2, p-ERK1/2, p-CREB proteins ↑ | **-** | Chen, Tang, et al. 2024 |
| Taohong Siwu Decoction | SD rat (-/male)  ·Bilateral surgical ligation of the common carotid arteries (2VO)  ·4.5 and 13.5 g/kg/d for 4 weeks (oral) | **Morris water maze test**  ·Escape latency ↓  ·Cross in target quadrant platform ↑  **Open-field test**  ·Time in the central region ↑  ·Total distance of activity ↓  **Novel object recognition test**  -Recognition index ↑ | **Hippocampus**  ·Neuronal atrophy or loss, cell border arrangement improved  ·Pyramidal cells restored  ·Neuronal apoptosis ↓  ·GRP78(Bip), p-PERK, p-IRE1α, elF2α, ATF6, ATF4 proteins ↓  ·Bcl-2 protein ↑ / Bax protein ↓ | - | Fan et al. 2024 |
| Zishen Huoxue Decoction | SD rat (-/male)  ·Permanent bilateral common carotid artery occlusion  ·8.9, 17.8, and 35.6 g/kg for 28 days (oral) | **Morris water maze test**  ·Escape latency ↓  ·Time in target quadrant platform ↑ | **Hippocampus (CA1)**  ·Neuron arrangement, atrophied cells, necrotic neurons recovered  ·Normal neurons ↑  ·Autophagosomes ↑  ·Mitochondrial swelling, permutation disorder, cristae decrease, mitochondrial vacuolation were improved  ·Mitochondrial membrane potential restored  ·Co-localization of Parkin and NeuN ↑  ·Parkin, PINK1, LC3-II/I proteins ↑  ·p62 protein ↓  ·Bax, caspase-3 proteins ↓ / Bcl-2 protein ↑  ·SOD ↑ / MDA ↓ | - | Zhao et al. 2024 |
|  | SD rat (8 weeks/male)  ·Permanent bilateral common carotid artery occlusion  ·half, normal, and twice of the adult dosage (intragastric) | **Morris water maze test**  ·Escape latency ↓  ·Cross in target quadrant platform ↑ | **Hippocampus**  ·Neuronal inflammation, atrophy, pyknosis, pyroptosis ↓  ·NVU IgG-positive cells, Iba-1^+^ microglia ↓, NeuN-labeled neurons ↑  ·LDH ↓  ·NLRP3, ASC, cleaved caspase-1, GSDMD-N proteins ↓  ·IL-1β, IL-18 proteins ↓  ·miR-124-3p ↑ | **NVU** (OGD-injured, medicated serum treated)  ·miR-124-3p ↑  ·Pyroptosis ↓  ·NLRP3, ASC, cleaved caspase-1, GSDMD-N proteins ↓  ·IL-1β, IL-18 proteins ↓  ·ROS, LDH ↓  ·BMECs, GFAP ↑ | Yao et al. 2023 |
| Wheat bran, citrus peel, jujube (WCJ, modified Gammaekdaejo decoction) | SD rat (8 weeks/male)  ·Bilateral common carotid artery occlusion  ·100 and 400 mg/kg for 30 days (-) | - | **Corpus callosum, optic tract (white matter)**  ·Damage severity scale ↓  ·GFAP-positive cells ↓  ·Iba1-positive cells ↓ | **Rattus norvegicus glioma cells** (hypoxic condition)  ·Cell survival ↑  ·Apoptotic cells ↓  -Bcl-2/Bax ratio ↑ | Kim, Lim, Hwang, and Lee 2024 |
| Banhabaekchulcheonma Tang | C57BL/6 mice (6 weeks/male)  ·Bilateral common carotid artery stenosis  ·40 and 80 mL/kg for 6 weeks | **Y-maze test**  ·No significant difference  **Novel object recognition test**  ·Distance traveled in zone 3 ↑  ·Discrimination ratio ↑ | **Cortex**  ·CD68^+^ microglia ↓  ·GFAP^+^ astrocytes ↓ | - | Kim, Lim, Cho, and Kim 2024 |
| Danggui Shaoyao San | SD rat (8 weeks/male)  ·Two-vessel occlusion of bilateral common carotid arteries  ·5 and 10 g/kg for 4 weeks (oral) | **Morris water maze test**  ·Escape latency ↓  ·Time in target quadrant platform ↑  ·Cross in target quadrant platform ↑ | **Hippocampus**  ·Neuronal damage (lost, shrinkage, disordered arrangement) ↓, neuronal apoptosis ↓  ·Bcl-2 protein ↑  ·Caspase-3 protein ↓  ·SYP, PSD95, HIF-1α, EPO, p-EPOR, p-STAT5, AKT1 proteins ↑ | **PC12 cells** (CoCl2-induced neurotoxicity)  ·Neuronal cell death, apoptosis ↓  ·Caspase-3 mRNA ↓  ·PSD95, HIF-1α, EPO, EPOR mRNAs ↑  · HIF-1α, EPO, p-EPOR/EPOR proteins ↑ | Yuan et al. 2024 |
| Buqi Huoxue Tongnao | SD rat (-/male)  ·Two-vessel occlusion  ·2.5, 5.0, and 10.0 g/kg/d for 30 days (oral) | **Open field test**  ·Number of fecal pellets ↓  **Y-maze test, Novel object recognition test**  ·Time exploring the novel arm and the novel object ↑ | **Hippocampus**  ·Nuclear condensation, neuronal loss, diseased cells ↓  ·PIK3CA, p-AKT/AKT mRNAs and proteins ↑  ·TNF, IL-1β mRNAs and proteins ↓  ·Caspase-3 mRNAs and proteins ↓  ·Bcl-2/Bax ↑  ·ROS, 8-OHdG, NO ↓  ·iNOS mRNA ↓  ·eNOS transcription ↑  **Cerebral blood flow** ↑ | **BV2 microglial cell** (OGD induced)  ·ROS and TNF-α ↓  **L02 cells** (OA/PA mediated lipid metabolism disturbances)  ·Red-positive area in the cytoplasm ↓  ·CYP7A1, LXRα mRNAs ↑  **Plasma**  ·TG, TC, LDL-C ↓  ·HDL-C, total protein, albumin ↑ | Gao et al. 2024 |
| Modiﬁed Dioscorea Pill | SD rat (-/male)  ·Bilateral common carotid artery occlusion  ·1 mL/100 g for 45 days (intragastric) | - | **Hippocampal CA1, CA3 and DG regions**  ·Neuronal damages recovered  ·Micro-vessel density ↑  ·Ang-1, Ang-2, Tie-2, VEGF mRNAs and proteins ↑ | - | Kuang et al. 2023 |
|  | SD rat (-/male)  ·Bilateral common carotid artery occlusion  ·1 mL/100 g for 25 days (intragastric) | **Morris water maze test**  ·Escape latency ↓  ·Time and cross in target quadrant platform ↑ | **Hippocampal CA1 region**  ·Neuronal arrangement, structural damage, nuclear condensation, presynaptic and postsynaptic membrane recovered  ·Pyramidal cells ↑  ·SYP, PSD-95, MAP-2 proteins ↑  ·CD43 labeled MVD ↓  ·GAP-43, VEGF mRNAs ↑ | - | Li et al. 2020 |
|  | SD rat (-/male)  ·Ligation of bilateral common carotid arteries  ·10 g/kg/d for 45 days (-) | **Morris water maze test**  ·Escape latency ↓  ·Time and distance in target quadrant platform ↑  ·Cross in target quadrant platform ↑ | **Hippocampal CA1 region**  ·Neuronal arrangement, structural damage, nervous fiber recovered  ·Pyramidal cells ↑  ·NF-kB p65, Bax, GFAP proteins ↓  ·MAP-2, Olig-2, Bcl-2 proteins ↑  ·GAP-43, VEGF mRNAs ↑ | - | Li 2019 |
| Modified Erchen Decoction | SD rat (-/male)  ·Bilateral common carotid arteries obstruction  ·2.5, 5.0, and 10.0 g/kg/d for 4 weeks (-) | **Open field test**  ·Vertical score ↑  **Y-Maze test**  ·Time entering the novel arm ↑ | **Hippocampus**  ·SOD, AOC ↑, MDA ↓  ·IL-6, IL-1β mRNAs ↓  ·Aβ42 ↓  ·JNK, Bax, p-JNK/JNK, JAK2, STAT3 mRNAs ↓  ·p-JAK2 positive cells ↓ | **BV2 microglial cell** (LPS stimulated; liquiritin, isochlorogenic acid C, hesperetin treated)  ·IL-6 ↓  ·JNK, JAK2 mRNAs ↓  **Plasma**  ·TG, LDL, Aβ42 ↓ | Gao et al. 2023 |
| Xi Xian Tong Shuan Capsule | SD rat (-/male)  ·Bilateral common carotid artery occlusion  ·500 mg/kg/d for 42 days (intragastric) | **Morris water maze test**  ·Escape latency ↓  ·Time and cross in target quadrant platform ↑  ·Swimming trajectories ↑ | **Cortex, Corpus callosum, striatum and hippocampus**  ·MBP, NF200 ↑  ·MAP2, NeuN ↑  **Cerebrospinal fluid**  ·IL-6, TNF-α, IL-33, MCP-1 ↓ | **Plasma**  ·IL-6, TNF-α ↓ | Yan et al. 2022 |
| Modified Daehwang Hwanglyoun Sasim Tang | C57BL/6 mouse (6 weeks/male)  ·Bilateral common carotid artery occlusion  ·5 and 50 mL/kg for 4 weeks (oral) | **Y-maze test**  ·Time taken for first-arm entry ↓ (N.S.)  ·Total number of times the mice into the three arms ↑ (N.S.)  ·Probability of sequentially entering the three arms ↑  **Novel object recognition test**  ·Total distance in the arena ↑ (N.S.)  ·Stay-time in zone 3, discrimination ratio ↑ | **Hippocampal CA1 region**  ·Cell density ↑  ·ERK, JNK proteins ↓  ·Mn-SOD ↓ | - | Lee et al. 2022 |
| Danshen Honghua herbal pair | Wistar rats (-/male)  ·Bilateral common carotid artery occlusion  ·3.2 g/kg/d for 28 days (intragastric) | **Morris water maze test**  ·Escape latency ↓  ·Time and cross in target quadrant platform ↑ | **Hippocampus**  ·Neuronal arrangement, structure restored  ·ACh ↑, AChE ↓  ·Nissle positive cells ↑  ·ROS ↓ | - | Zhang et al. 2022 |
| Shenzhi Jiannao Formula Granule | SD rat (-/male)  ·Permanent bilateral common carotid artery occlusion  ·0.2 g/kg for 14 days (intragastric) | **Morris water maze test**  ·Escape latency, total distance ↓  ·Time in target quadrant platform ↑  **Novel object recognition test**  -Discrimination index ↑ | **Hippocampal CA1 region**  ·Neuronal arrangement, loss, damage recovered  ·Clathrin, RAB5B proteins ↑  ·Activation of NMDAR1 ↓  ·Cytoplasmic NMDAR1 protein ↑  ·Clathrin mRNA ↑, NMDAR1 mRNA ↓ | **PC12 cell**  (Glutamate induced damage)  ·Number and viability of cells ↑  ·LDH ↓  .Clathrin, RAB5B proteins ↑, NMDAR1 protein ↓  ·Clathrin mRNA ↑, NMDAR1 mRNA ↓ | Tian et al. 2021 |
| Bushen Huoxue Formula | SD rat (8 weeks/male)  ·Ligation of common carotid artery  ·10.14 g/kg/d for 28 days (intragastric) | **Morris water maze test**  ·Escape latency ↓  ·Number of times crossed the original platform ↑ | **Hippocampal CA1 area**  ·Cell structure, nuclei, neuronal damage recovered  ·Cell number, Nissl bodies ↑  ·Beclin-1, LC3 ↓, P62 ↑ | - | Luo et al. 2021 |
| Bushen Huoxue Decoction | Wistar rat (-/-)  ·Bilateral common carotid artery occlusion  ·2.75, 5.5, and 11 g/kg for 14 days (intragastric) | **Morris water maze test**  ·Escape latency ↓  ·Cross in target quadrant platform ↑ | **Hippocampal CA1 and parietal cortex**  ·Synapse density reversed  ·MDA ↓, SOD, CAT, GSH ↑  ·GPx-1, CAT proteins and mRNAs ↑ | - | Ye et al. 2014 |
| Nao Tai Fang | SD rat (-/male)  ·Bilateral common carotid artery ligation  ·34 g/kg for 30 days (intragastric) | **Morris water maze test**  ·Escape latency ↓  ·Cross in target quadrant platform ↑ | **Hippocampus**  ·Neuron pyramidal cells neatly arranged, cell gap smaller, cell nucleus increased  ·IL-1β, TNF-α proteins and mRNAs ↓ | - | Zhao et al. 2021 |
| Modiﬁed Buyang Huanwu Decoction | SD rat (-/male)  ·Bilateral common carotid artery ligation  ·17.0, 34.0, and 51.0g/kg for 30 days (intragastric) | **Morris water maze test**  ·Escape latency ↓  ·Number of times crossed the platform ↑ | **Hippocampus**  ·Neuron pyramidal cells neatly arranged, outline cleared  ·Inﬂammatory cell inﬁltration ↓  ·Bcl-2-positive cells ↑, Bax- and caspase-3-positive cells ↓  ·SIRT1, IκBα proteins and mRNAs ↑  ·NF-κB p65 protein and mRNA ↓  ·Bcl-2 protein and mRNA ↑, Bax and caspase-3 proteins and mRNAs ↓ | - | Yang et al. 2021 |
| Wuzang Wenyang Huayu Decoction | SD rat (-/male)  ·Bilateral carotid arteries occlusion  ·2.5 g/kg for 2 weeks (-) | **Morris water maze test**  ·Escape latency ↓  ·Distance ratios ↑ | **Hippocampus**  ·Tissue injury, condensed nuclei, neuronal plasticity recovered | - | [S1] |
| Yi Gan San Granule | SD rat (8 weeks/male)  ·Bilateral occlusion of the common carotid arteries  ·1 g/kg/d for 22 days (oral) | **Morris water maze test**  ·Escape latency ↓  ·Time and cross in target quadrant platform ↑ | **Hippocampus CA1 region**  Histology:  ·Neuronal damage, karyopyknosis ↓  ·GSH-Px and SOD ↑, MDA ↓ | - | Liao et al. 2020 |
| Dengzhan Shengmai Capsule | SD rat (-/male)  ·Permanent bilateral occlusion of the common carotid arteries  ·0.72 g/kg/d for 4 weeks (oral) | **Morris water maze test**  ·Escape latency ↓  ·Time of first arrival on platform ↓  ·Effective residence time ↑ | **Hippocampal CA1 region**  ·Neuronal shrinkage and loss improved  ·SOD ↑, MDA ↓ | **SH-SY5Y cells**  (4,5 caﬀeoylquinic acid, scutellarin pretreated)  ·GABRA1 ↑, NMDA NR2B ↓ | Sheng et al. 2020 |
| Hwangryunhaedok Tang | SD rat (-/male)  ·Bilateral common carotid artery occlusion  ·200 and 400 mg/kg (for commercial granule); 400 mg/kg (for extract) for 30 days (oral) | **Y-maze test**  ·Spontaneous alternation ↑  **Novel object recognition test**  ·Discrimination percentage for the novel object ↑ | **Hippocampal CA1, CA3 regions**  ·Surviving cells ↑  ·ACh ↑, AChE ↓  ·Anti-Iba-1 cells ↓  Protein expression:  ·JNK, p-p38 MAPK proteins ↓ | - | Sohn et al. 2019 |
| Choto San | SD rat (-/male)  ·Common carotid artery occlusion  ·375 and 750 mg/kg for 4 weeks (intragastric) | **Morris water maze test**  ·Escape latency ↓  ·Total time and distance in target quadrant and platform site crossing ↑ | **Hippocampal CA1, CA3 and dentate gyrus regions**  ·Neuronal atrophy, neuronal loss recovered  ·Caspase-3 activity, ROS level ↓  ·Iba-1 protein ↓  ·Total and nuclear Nrf2 proteins ↑, cytosol Nrf2 protein ↓  ·NQO1, HO-1 proteins ↑ | - | Jiang et al. 2019 |
|  | ICR mouse (-/male)  ·Permanent occlusion of common carotid arteries  ·750 mg/kg/d for 5 days (oral) | **Object recognition test-1 and -2**  ·Exploring time to novel object ↑  **Object location test**  ·Exploration times for an object in a novel location enhanced  **Object context test**  ·Discriminating between the old and target objects | **Cerebral cortex and hippocampus**  mRNA expression:  ·ChAT mRNA ↑  ·Muscarinic m3-, m5-receptor mRNAs ↑ | - | Zhao et al. 2007 |
|  | Wistar rat (-/male)  ·Permanent occlusion of bilateral common carotid arteries  ·75–225 mg/kg for 4 days (oral) | - | **Cortex and hippocampus**  ·M-CSF, M-CSF receptor mRNAs ↑ | **C6Bu-1 glioma cells**  · M-CSF mRNA ↑ | Obi et al. 2007 |
|  | ICR mouse (-/male)  ·Common carotid arteries occlusion  ·Choto-san 750 mg/kg; Uncaria-free Choto-san 337.5 and 675 mg/kg; Uncaria alone 37.5 and 75 mg/kg for 2 weeks (oral) | **Water maze test**  ·Learning performance in training trials improved (Uncaria)  **Tube-dominance test**  ·Dominance behavior ↓ (Choto-san) | **Cerebral cortex and hippocampus**  ·ACh ↑ (Choto-san, Uncaria) | - | Murakami et al. 2005 |
| Xiaoshuan Enteric-Coated Capsule | SD rat (-/male)  ·Bilateral common carotid artery occlusion  ·47, 140, and 420 mg/kg for 40 days (oral) | **Morris water maze test**  ·Faster platform finding time  ·Escape latency and path length in hidden platform ↓  ·Path and time in previous platform ↑ | **Hippocampal CA1 region**  ·Axon swelling and myelin degeneration improved  ·Synaptic and postsynaptic density, length, thickness ↑  ·Surviving cells, NeuN-positive neurons ↑  ·Hippocampal CBF ↑  ·Signal intensities of bilateral ACA and ICA ↑, tortuosity of BA ↓  ·^18^F-FDG accumulation ↑  ·APP, p-CRMP2 proteins ↓  ·SYP, GAP-43, p-AKT, p-GSK-3β proteins ↑ | - | Li et al. 2018 |
| Daming Capsule | SD rat (-/male)  ·Ligation of bilateral common carotid arteries  ·100 and 200 mg/kg for 30 days (oral) | **Morris water maze test**  ·Escape latency ↓  ·Swimming distance in the target quadrant ↑ | **Cerebral cortex and hippocampus**  ·Neuronal cell loss, nuclei shrinkage, glial proliferation recovered  ·MDA, SOD ↓  ·GPx, CAT ↑ | - | Song et al. 2015 |
| Kaixin Jieyu Prescription | SD rat (7 weeks/male)  ·Permanent ligation of bilateral common carotid arteries  ·0.45, 0.9, and 1.8 g/kg/day 21 days (-) | **Sucrose preference test**  ·Sucrose preference ↑  **Open-field test**  ·Moving distance and number of rearing ↑ | **Cortex and hippocampus**  ·CBF ↑  ·Apoptotic rate ↓  ·BDNF, TrkB, t-PA proteins and mRNAs ↑  ·ZO-1, occludin, claudin-5 proteins ↑  ·PAI-1, MMP-2 proteins and mRNAs ↓ | - | Pan et al. 2015 |
| Cardiotonic Pill | Wistar rat (12 weeks/male)  ·Permanent bilateral common carotid artery occlusion  ·200 mg/kg for 22 days (oral) | - | **White matter and hippocampus**  ·MBP, Iba-1-positive cells, OX-6 positive cells ↑  ·COX-2, IL-1b, IL-6, p-ERK/ERK, p-p38/p38 ↓ |  | Lee et al. 2013 |

8-OHdG, 8-hydroxy-2′-deoxyguanosine; ^18^F-FDG, fluorodeoxyglucose F18; ACA, anterior cerebral artery; ACh, acetylcholine; AChE, acetylcholinesterase; AKT, protein kinase B; Ang, angiopoietin; AOC, antioxidant capacity; APP, amyloid precursor protein; ASC, apoptosis-associated speck-like protein containing a caspase recruitment domain; ATF, activating transcription factor; BA, basilar artery; Bax, Bcl-2 associated X protein; Bcl-2, B cell lymphoma-2; BDNF, brain-derived neurotrophic factor; BMEC, brain microvascular endothelial cells; ChAT, choline acetyltransferase; COX-2, cyclooxygenase-2; CREB, cAMP response element-binding protein; CRMP2, collapsin response mediator protein-2; CYP, cytochrome P450; eIF2α, eukaryotic initiation factor2α; eNOS, endothelial nitric oxide synthase; EPO, erythropoietin; GABRA1, gamma-aminobutyric acid type A receptor subunit alpha1; ERK1/2, extracellular Signal-Regulated Kinase 1/2; GAP-43, growth-associated protein 43; GFAP, glial fibrillary acidic protein; GluA, glutamate ionotropic; GRP78(Bip), binding immunoglobulin protein; GSDMD-N, gasdermin D N-terminal fragment; GSK-3β, glycogen synthase kinase-3β; HDL-C, high-density lipoprotein cholesterol; HIF-1, hypoxia-inducible factor-1; HO-1, heme oxygenase 1; ICA, internal carotid artery; iNOS, inducible nitric oxide synthase; IL, interleukin; Iba1, ionized calcium binding adapter molecule 1; IRE1α, inositol-requiring transmembrane kinase/endoribonuclease 1α; LC3, light chain 3; LDH, lactate dehydrogenase; LDL-C, low-density lipoprotein cholesterol; LXRα, liver X receptor alpha; MAP-2, microtubule-associated protein-2; MBP, myelin basic protein; MDA, malondialdehyde; MCP-1, monocyte chemoattractant protein-1; MEK1/2, mitogen-activated Protein Kinase 1 and 2; MMP-2, matrix metalloproteinase-2; Mn-SOD, manganese superoxide dismutase; MVD, micro vessel density; NeuN, neuronal nuclei; NF200, neurofilament 200; NMDAR, N-methyl-d-aspartic acid receptor 1; NLRP3, nucleotide oligomerization domain-like receptors 3; NO, nitric oxide; NQO1, NAD(P)H:quinone oxidoreductase 1; Nrf2, nuclear factor-E2-related factor 2; NVU, neurovascular unit; OA, oleic acid; OGD/R, oxygen-glucose deprivation/reperfusion; Olig-2, oligodendrocyte transcription factor 2; OX-6, mouse monoclonal anti-MHC class II RT1B antibody; PA, palmitic acid; PAI-1, plasminogen activator inhibitor-1; PERK, protein kinase R (PKR)-like endoplasmic reticulum kinase; PI3K, phosphatidylinositol-3-kinase; PSD-95, postsynaptic density protein 95; PKA, protein kinase A; ROS, reactive oxygen species; SOD, superoxide dismutase; STAT, signal transducers and activators of transcription; SYP, synaptophysin; TC, total cholesterol; Tie-2, tunica interna endothelial cell kinase-2; TG, triglyceride; TNF, tumor necrosis factor; t-PA, tissue plasminogen activator; TrkB, tropomyosin receptor kinase B; VEGF, vascular endothelial growth factor; ZO-1, zona occludens protein-1.

**Table S3**. Pharmacological effects of single herbal medicines in animal model of VaD (ordered by publication year)

| **Material** | **Animal (age/sex)**  **·Model**  **·Administration (route)** | **Cognitive ability** | **Organ** | ***in vitro* / Blood** | **Ref.** |
| --- | --- | --- | --- | --- | --- |
| *Gardenia jasminoides* fruit | C57BL/6 mouse (-/-)  ·Bilateral common carotid artery stenosis  ·50 mg/kg for 28 days (intragastric) | **Morris water maze test**  ·Escape latency ↓  ·Number of platform crossing ↑  ·Latency to find the platform ↓  ·Duration in the target quadrant ↑  **Y-maze test**  ·Alternation accuracy ↑ | **White matter**  ·Corpus callosum and Myelin sheaths arrangement recovered  ·MBP, MAG, MOG proteins ↑  ·MDA ↓, SOD, GSH ↑  ·Keap1 protein ↓, Nrf2, HO-1 proteins ↑  ·PLA2, SMPD1, SMPD2 mRNAs ↓, Sgms1 mRNA ↑  ·TUNEL-positive oligodendrocytes ↓  ·Bcl-2/Bax ratio ↑ | **OLN-93 cells** (H_2_O_2_ induced; crocetin treated)  ·Apoptosis rate ↓  ·Bcl-2/Bax ratio ↑  ·Caspase-3 ↓  ·ROS ↓  ·Nrf2 mRNA ↓  ·Translocation of Nrf2 to nucleus  **Serum**  ·MDA ↓, SOD, GSH ↑  ·CBF ↑ | Pang et al. 2024 |
|  | SD rat (-/male)  ·Bilateral common carotid arteries occlusion in hyperlipidemic rats  ·10 and 50 mg/kg for 3 weeks (oral) | **Morris water maze test**  ·Time to first reach the platform ↓  ·Time in the target quadrant ↑  ·Number of platform crossing ↑  **Step-down test**  ·Time staying on the platform ↑  ·Number of errors ↓ | **Cortex and hippocampus CA1 region**  ·Number of neurons, Nissl-stained cells ↑  ·Neuronal damages (vacuolated cytoplasm, pyknotic nucleus) recovered  ·TNF-α, IL-1β mRNAs ↓  ·TGF-β, IL-4 mRNAs ↑  ·Nuclear PPAR-γ protein ↑  ·NF-κB protein ↓, C/EBPβ protein ↑  **Microglial cell**  ·CD11b-positive cells ↓  M2 markers (Arg1, Ym1 mRNAs) ↑  M1 markers (CD68, CD86 mRNAs) ↓ | **Serum**  ·TC, TG, LDL-C ↓  ·CBF ↑ | Liu et al. 2022 |
| *Ligusticum chuanxiong* Rhizome | C57BL/6 mouse (8 weeks/male)  ·Bilateral common carotid artery stenosis  ·5, 50, and 500 mg/kg (oral) | **Novel object recognition test**  ·Exploration time in zone 3 ↑  ·Ratio of time in zone 2 and zone 3 ↑  **Y-maze test**  ·No difference | **White matter**  ·GFAP-positive cells, CD68-positive cells ↓  ·p-ERK, p-p38, p-JNK proteins ↓  ·Wnt3, p-GSK3β proteins ↓ | - | Lim et al. 2024 |
| *Perilla frutescens* leaves | SD rat (8 weeks/male)  ·Two-vessel occlusion and hypovolemia  ·30, 60, and 90 mg/kg for 23 days (oral) | **Y-Maze test**  ·Spontaneous alternations spared  **Barnes Maze test**  ·Moving distance to the goal ↓  ·Time in the target quadrant ↑  **Passive Avoidance test**  ·Escape latencies ↑ | **Hippocampus CA1 region**  ·Pyramidal neurons ↑  ·Iba1^+^ microglia ↓ | **BV-2 cells** (LPS-stimulated)  ·NO, TNF-α, IL-6 ↓  ·p-p38, p-ERK1/2, and p-JNK, iNOS, NFκB ↓ | Kang et al. 2022 |
| Epimedium extract | C57BL/6 mouse (10-12 weeks/male)  ·Bilateral common carotid artery occlusion  ·50, 100, and 200 mg/kg/d for 8 weeks (intragastric) | **Morris water maze test**  ·Escape latency ↓  ·Time in the target quadrant ↑  ·Number of platform crossing ↑ | **Hippocampus**  ·Iba-1, GFAP ↓  ·TNF, IL1β, IL6, MMP-9 mRNAs ↓  ·AKT mRNA ↑  ·p-ERK1/2, p-JNK1/2, p-p38MAPK mRNAs ↓ | - | Xie et al. 2022 |
| Epimedium ﬂavonoids | SD rat (2 months/male)  ·Permanent bilateral common carotid artery ligation  ·50, 100, and 200 mg/kg/d for 12 weeks (intragastric) | **Novel object recognition test**  ·Discrimination index ↑  **Y maze test**  ·Percentage of alternation behavior ↑ | **Hippocampal CA1 region and cerebral cortex**  ·NeuN-positive cells ↑  **Hippocampus**  ·Synapse ultra-structure protected  ·Number of synapses ↑  ·MAP-2-labeled neuronal dendrites recovered  ·Bax, caspase-3 proteins ↓, Bcl-2 protein ↑  ·MAP-2, NF-200 proteins ↑  ·SYP, SYT1, SYN1, PSD-95 proteins ↑  ·Phosphorylations of NMDA2B and CaMKII-α proteins ↑  ·NRG-1, p-ErbB4, BDNF, p-Fyn proteins ↑  ·PI3K subunits p85 and p110α, p-Akt, p-CREB proteins ↑ | - | Niu, Ma, et al. 2020 |
|  | SD rat (-/male)  ·Permanent bilateral common carotid artery ligation  ·50, 100, and 200 mg/kg/d for 12 weeks (intragastric) | **Morris water maze test**  ·Escape latency ↓  ·Time in the target quadrant ↑  ·Number of platform crossing ↑  **Step-through test**  ·Latency ↑  ·Number of errors ↓ | **Cerebral white matter**  ·Demyelination, axon injury ↓  ·FA, ADC, RD values ↑  ·Myelin density, mature oligodendrocytes ↑  ·MBP protein ↑  ·Lingo-1, ROCK2 proteins ↓, p-Fyn protein ↑  ·BDNF, TrkB, NRG-1, p-ErbB4, PI3K p85 and p110α, p-Akt, p-CREB proteins ↑ | - | Niu, Wang, et al. 2020 |
| Broussonetiae Fructus | SD rat (-/male)  ·Permanent bilateral common carotid artery ligation  ·For 42 days (intragastric) | **Morris water maze test**  ·Escape latency ↓  ·Frequency of platform crossing ↑ | **Cortex and hippocampus**  ·Co-localization of MAP2 and synaptophysin with NeuN normalized  ·SYP protein ↑  ·Iba-1 protein ↓  ·iNOS, CD16 proteins ↓  ·Arginase-1 protein ↑  **White matter**  ·MBP ↑ | - | Liu, Wang, et al. 2020 |
| *Panax ginseng* | Wistar rat (4–5 months/-)  ·Middle cerebral artery occlusion  ·50 and 100 mg/kg for 8 days (intragastric) | **Morris water maze test**  ·Escape latency ↓  ·Percent distance in the target quadrant ↑  **Balance beam test**  ·No differences in traversal time | **Hippocampus**  ·Degenerating and necrotic neurons ↓  ·GFAP-immunoreactive cells ↓  ·AChE activity ↓, ChAT activity ↑  ·VEGF, bFGF proteins ↑  ·Bcl-2 ↑, Bax ↓ | - | Zhu et al. 2018 |
| *Scutellaria baicalensis* | SD rat (19 months/male)  ·Bilateral common carotid artery occlusion  ·**Stem-leaf total ﬂavonoids**: 50 and 100 mg/kg/d for 60 days (intraperitoneal) | **Morris water maze test**  ·Escape latency ↓  ·Searching time for the platform ↑  ·Percentage of traveled distance in the target quadrant ↑ | **Cortex and hippocampus**  ·p-tau (tau Ser199, tau Ser202, tau Thr205, tau Ser396, tau Ser404) proteins ↓  ·GSK3βser9 protein ↑  ·p35, p25 proteins ↓  ·PP2A-sub B protein ↑ | - | Cao et al. 2016 |
|  | Wistar rat (12 weeks/male)  ·Permanent occlusion of the bilateral common carotid artery  ·**Root extract**: 100 and 200 mg/kg/d for 40 days (oral) | **Spatial memory test**  ·Accuracy of spatial learning in the water maze ↑ | **Hippocampus**  ·p-ERK protein ↓  ·p-JNK, p-p38 proteins ↑ | - | Hwang et al. 2011 |
| *Ginkgo biloba* leaves | Wistar rat (-/male)  ·Permanent bilateral common carotid artery occlusion  ·5, 10, 20, and 40 mg/kg/d for 42 days (oral) | - | **Hippocampus and white matter**  ·Microglial proliferation, astrocytes ↓  **Hippocampus**  ·TNF-α, IL-1β, IL- 6, TLR4 proteins ↓  ·MyD88, RAGE, Ang-II proteins ↓  ·p-ERK, p-p38 ↓  **Medial septum**  ·ChAT-positive cells ↑ | - | Kim et al. 2016a |
|  | Mongolian gerbils (-/male)  ·Bilateral occlusion of the common carotid arteries  ·60 and 100 mg/kg/d for 25 days (oral) | **Object location test**  ·Discrimination between the new and the familiar location of the object | Hippocampus CA1  ·Neuronal loss ↓  ·GFAP immunoreactivity ↓ | **Blood**  ·SOD ↑ | Rocher et al. 2011 |
| Mume Fructus | Wistar rat (12 weeks/male)  ·Permanent bilateral common carotid artery occlusion  ·200 mg/kg for 22 days (oral) | - | **White matter and hippocampus**  ·GFAP, Iba-1-positive microglia ↓  **Hippocampus**  ·Cox-2, IL-1b, IL-6 proteins ↓  ·Ang-II, RAGE proteins ↓  ·p-ERK, p-p38 proteins ↓  ·ChAT positive cells, ChAT protein ↑ | - | Kim et al. 2016b |
|  | Wistar rat (12 weeks/male)  ·Permanent bilateral common carotid artery occlusion  ·200 mg/kg for 21 days (oral) | - | **White matter and hippocampus**  ·Neuronal cell death ↓  ·MBP ↑  ·Iba-1-positive cells, GFAP-positive cells ↓  ·COX-2, IL-1β, IL-6 proteins ↓  ·TLR4, MyD88, p-p38 MAPK proteins ↓ | - | Lee et al. 2015 |
|  | Wistar rat (12 weeks/male)  ·Permanent bilateral common carotid artery occlusion  ·100, 200, and 400 mg/kg/d for 42 days (oral) | **Morris water maze test**  ·Time in target counter ↑ | **Hippocampus**  ·Iba-1 positive cells, OX-6 positive cells ↓  ·p-ERK1/2 protein ↓  ·Cytosolic IκB-α and NF-κB p65 proteins ↑, nuclear NF-κB p65 protein ↓ | - | Jeon et al. 2012 |
| *Salvia miltiorrhiza* | Wistar rat (12 weeks/male)  ·Permanent occlusion of the bilateral common carotid artery  ·200 mg/kg for 22 days (oral) | - | **White matter and hippocampal CA1 region**  ·Iba-1 positive microglia ↓  ·MBP ↑  ·TLR4, MyD88 proteins ↓  **Hippocampus**  ·TNF-α, IL-1β, IL-6 proteins ↓ | - | Kim et al. 2015 |
| Cistanche glycosides | Wistar rat (6 months/male)  ·Bilateral common carotid artery ligation  ·10 mg/kg/d for 14 days (intraperitoneal) | **Morris water maze test**  ·Escape latency ↓ | **Hippocampus**  ·TXNL 1, MAPKK 1, DPYSL2 proteins ↑  ·GL protein ↓  ·p-tau protein ↓ | - | Chen et al. 2015 |
| *Angelica sinensis* root | Wistar rat (-/male)  ·Cranial irradiation (10 Gy) + permanent bilateral common carotid artery occlusion  ·1 g/kg/d for 28 days (-) | **Morris water maze test**  ·Escape latency ↓  ·Time spent in platform region ↓ | **Hippocampus**  ·BrdU-incorporated cells ↓  ·Ratio of BrdU+ /NeuN+ cells ↑  ·SYP, PSD-95 proteins ↑  ·BDNF, p-CREB proteins ↑  ·GAD65 signal density ↑ | - | Xin et al. 2013 |
|  | Wistar rat (10 weeks/male)  ·Permanent bilateral common carotid artery occlusion  ·Injection (50 g/100mL): 15 mL/kg/d for 8 weeks (intravenous) | **Water maze test**  ·Escape latency ↓  ·Time in the quadrant Q1 ↑ | **Hippocampus**  ·NGF mRNA ↑  ·No differences in BDNF mRNA  ·NGF, BDNF proteins ↑ | - | Zheng et al. 2008 |
| Saffron (*Crocus sativus* L.) | Wistar rat (-/male)  ·Permanent occlusion of the common carotid arteries  ·50, 100, and 250mg/kg/day for 5 days (intraperitoneal) | **Morris water maze test**  ·Escape latency ↓  ·Swimming time in the target quadrant ↑  ·Traveled distance to platform ↓ | - | - | Hosseinzadeh et al. 2012 |
| *Triticum aestivum* L. | SD rat (-/male)  ·Bilateral common carotid artery occlusion  ·400 mg/kg/day for 7 days (oral) | **Morris water maze test**  ·Escape latency ↓ | - | - | [S2] |

AChE, acetylcholinesterase; ADC, apparent diﬀusion coeﬃcient; AKT, protein kinase B; Ang, angiopoietin; Bax, Bcl-2 associated X protein; Bcl-2, B cell lymphoma-2; BDNF, brain-derived neurotrophic factor; bFGF, basic fibroblast growth factor; CaMKII-α, calcium/calmodulin-dependent protein kinase II; C/EBPβ, CCAAT/enhancer binding protein beta; ChAT, choline acetyltransferase; CREB, cAMP response element-binding protein; DPYSL2, dihydropyrimidinase-related protein 2; ErbB4, Erb-b2 receptor tyrosine kinase 4; ERK1/2, extracellular Signal-Regulated Kinase 1/2; FA, fractional anisotropy; GAD65, glutamate Decarboxylase 65; GAP-43, growth-associated protein 43; GCL, glutathione synthetase; GFAP, glial fibrillary acidic protein; GSK-3β, glycogen synthase kinase-3β; HO-1, heme oxygenase 1; iNOS, inducible nitric oxide synthase; IL, interleukin; Iba1, ionized calcium binding adapter molecule 1; JNK, c-Jun amino N-terminal kinases; keap1, Kelch-like ECH associated protein 1; LDL-C, low-density lipoprotein cholesterol; MAG, myelin-associated glycoprotein; MAP-2, microtubule-associated protein-2; MAPK, mitogen-activated protein kinase; MAPKK 1, mitogen-activated protein kinase kinase 1; MBP, myelin basic protein; MDA, malondialdehyde; MMP-9, matrix metalloproteinase-9; MOG, myelin oligodendrocyte glycoprotein; MYD88, myeloid differentiation primary response 88; NeuN, neuronal nuclei; NF-κB, nuclear factor kappa B; NGF, nerve growth factor; NMDAR, N-methyl-d-aspartic acid receptor 1; NO, nitric oxide; Nrf2, nuclear factor-E2-related factor 2; NRG-1, neuregulin-1; OX-6, mouse monoclonal anti-MHC class II RT1B antibody; PI3K, phosphatidylinositol-3-kinase; PLA2, phospholipase A2; PSD-95, postsynaptic density protein 95; PP2A, protein Phosphatase 2A; PPAR-γ, peroxisome proliferator-activated receptor-gamma; RAGE, receptor for advanced glycation endproducts; RD, radial diﬀusivity; ROCK2, Rho associated coiled-coil containing protein kinase 2; ROS, reactive oxygen species; Sgms, sphingomyelin synthase; SMPD1, acid sphingomyelinase 1; SMPD2, sphingomye-linase phosphodiesterase 2; SOD, superoxide dismutase; SYN1, synapsin 1; SYP, synaptophysin; SYT1, synaptotagmin 1; TC, total cholesterol; TG, triglyceride; TGF-β, transforming growth factor-β; TNF, tumor necrosis factor; TrkB, tropomyosin receptor kinase B; TXNL 1, thioredoxin-like protein 1; VEGF, vascular endothelial growth factor.

**Table S4**. Pharmacological effects of phytochemicals in animal model of VaD (ordered by publication year)

| **Material** | **Animal (age/sex)**  **·Model**  **·Administration (route)** | **Cognitive ability** | **Organ** | ***in vitro* / Blood** | **Ref.** |
| --- | --- | --- | --- | --- | --- |
| Gastrodin (from *Gastrodia elata* Blume) | SD rat (-/male)  ·Bilateral common carotid arteries occlusion  ·25 and 50 mg/kg for 8 weeks (intragastric) | **Morris water maze test**  ·Escape latency ↓  ·Residence time and platform crossing times in the target quadrant ↑ | **Hippocampus**  ·Neuronal necrosis and pyknotic nuclei recovered  ·Mitochondrial swelling, fragmentation, matrix disruption, compromised or absent cristae restored  ·Mitochondrial ATP production ↑  ·Mitochondrial NADH, NDUFB8, SDHB, UQCRC2, MTCO1, ATP5A ↑  ·Fis1, DRP1 proteins ↓  ·TFAM, Mfn1/2 proteins ↑  ·GSH, SOD ↑  ·P16, P21, P53 proteins ↓  ·SIRT3 ↑ | **HT22 cells** (hypoxia-induced)  ·LDH, mtROS ↓  ·GSH, SOD ↑  ·ATP ↑  ·MMP ↑, mitochondrial fragmentation ↓  ·NDUFB8, SDHB, UQCRC2, MTCO1, ATP5A ↑  ·Fis1, DRP1 proteins ↓  ·Mfn1/2 proteins ↑  ·mtDNA-specific TFAM protein ↑  ·TFAM acetylation ↓  ·NDUFS8, UQCRC1 genes ↑  ·HO-1, Prdx1 proteins ↑  ·P16, P21, P53 proteins ↓  ·SIRT3 ↑    **Serum**  ·GSH, SOD ↑ | Chen, Yang, et al. 2024 |
|  | SD rat (-/male)  ·Bilateral common carotid arteries occlusion  ·25 and 50 mg/kg for 8 weeks (intragastric) | **Morris water maze test**  ·Escape latency ↓  ·Crossing times in the target quadrant ↑  ·Time in the target quadrant ↑ | **Hippocampus**  ·Neuronal arrangement, pyknotic nuclei, degeneration, characteristic necrosis recovered  ·Neuronal apoptosis ↓, Bcl2 protein ↑, Bax protein ↓  ·LC3II/LC3I, Beclin-1 proteins ↓  ·P62 protein ↑  ·p-PI3K/PI3K, p-AKT/ AKT proteins ↑ | **HT22 cells** (hypoxia-induced)  ·Apoptosis ↓, Bcl2 protein ↑, Bax protein ↓  ·LC3II/LC3I, Beclin-1 proteins ↓  ·P62 protein ↑  ·p-PI3K/PI3K, p-AKT/ AKT proteins ↑ | Chen et al. 2023 |
|  | SD rat (-/male)  ·Bilateral common carotid arteries occlusion  ·25, 50, and 100 mg/kg for 8 weeks (-) | **Morris water maze test**  ·Escape latency ↓  ·Dwell time in the original target quadrant ↑  ·Crossing times in the target quadrant ↑ | **Hippocampus**  ·Fe^2+^, MDA ↓, GSH ↑  ·GPx4 protein ↑  ·COX-2 protein ↓  ·Nrf2 protein ↑, Keap1 protein ↓ | **HT22 cells** (hypoxia-induced ferroptosis)  ·GPx4 protein ↑  ·COX-2 protein ↓  ·ROS ↓, GSH ↑  ·Fe^2+^ ↓  ·Nrf2 protein ↑, Keap1 protein ↓ | Li et al. 2022 |
|  | SD rat (-/male)  ·Bilateral common carotid arteries occlusion  ·22.5 and 90 mg/kg for 4 weeks (intragastric) | **Morris water maze test**  ·Escape latency ↓  ·Crossing times in the target quadrant ↑  ·Time searching for the target quadrant ↑  **Attention set shift test**  ·Times to reach criterion performance ↑ | **Hippocampal CA1 region**  ·Neuronal structure, density, alignment improved  ·p-Tau protein, Aβ_1–40/42_, Aβ_1–42_ proteins ↓ | **Plasma**  ·Aβ_1–42_ protein ↓ | Shi et al. 2020 |
|  | SD rat (-/male)  ·Bilateral common carotid arteries occlusion  ·15, 30, and 60 mg/kg for 28 days (oral) | **Morris water maze test**  ·Escape latency ↓  ·Time in the target quadrant ↑ | **Hippocampal CA1 and CA3 region**  ·Neuronal loss, shrinkage, cellular edema recovered  ·Apoptosis ↓  ·Aβ_1–40/42_ protein ↓  ·APP, BACE1 proteins ↓  ·ADAM10, IDE proteins ↑  ·Beclin-1, LC3-I proteins ↓  ·p62 protein ↑  ·p-P38 MAPK, Bax proteins ↓, Bcl-2 protein ↑ | **-** | Liu et al. 2018 |
| Gastrodin, Gastrodigenin | SD rat (-/male)  ·Bilateral common carotid artery ligation  ·25 and 50 mg/kg for 28 days (oral) | **Morris water maze test**  ·Escape latency ↓  ·Time searching for the target quadrant ↑  ·Time in the target quadrant ↑  ·Step-through latency ↑ | Hippocampal CA1, CA3 regions  ·Neuronal loss, degeneration, pyknotic nuclei, severe cellular edema recovered  ·Aβ_1-42_, p-tau396, p-tau217 proteins ↓ | **HT-22 cells** (H_2_O_2_-treated)  ·Mitochondrial respiration ↓  ·Basal respiration, maximal respiration, ATP production ↑ | Wu et al. 2023 |
| Schisandrin B | SD rat (-/male)  ·Bilateral common carotid artery occlusion  ·10, 20, and 40 mg/kg for 14 days (oral) | **Morris water maze test**  ·Escape latency ↓  ·Time in the target quadrant ↑ | **Hippocampus**  ·Pyramidal cells, cell nuclei ↑  ·HIF-1α, TRAK2, HUMMR proteins ↓ | - | He et al. 2024 |
| Mumefural | C57BL/6 male (8 weeks/mice)  ·Unilateral common carotid artery occlusion  ·40 mg/kg for 2 months (oral) | **Y-maze test**  ·Non-significant differences  **Open ﬁeld test**  ·No obvious defects in locomotion or anxiety  **Novel object test**  ·Recognition index ↑  **Novel location recognition test**  ·No influence on motivation or innate exploratory behavior | **Hippocampus (Ipsilateral)**  ·Ipsilateral hippocampus weight ↑  ·GLUT1, GLUT3, AChE proteins ↓  ·BDNF protein ↑  ·p-CREB/CREB, p-ERK/ERK ↑ | - | Kim et al. 2023 |
|  | Wistar rat (12 weeks/male)  ·Bilateral common carotid artery occlusion  ·20, 40, and 80 mg/kg for 42 days (oral) | **Morris water maze test**  ·Escape latency ↓ | **Basal forebrain**  ·ChAT-positive cells ↑  ·ChAT, VAChT proteins ↑, AChE protein ↓  **Hippocampus**  ·ChAT, VAChT proteins ↑, AChE protein ↓  ·PSD-95, SYP-1 proteins ↑  ·NMDAR2A, NMDAR2B proteins ↑  ·p-CaMKII protein ↑  ·BDNF, p-CREB/CREB proteins ↑  ·P2X7R, TLR4, MyD88 proteins ↓  ·NLRP3, caspase-1, IL-1β, IL-18 proteins ↓  ·NF-κB (p65, p50) proteins ↓  ·p-STAT 3 Tyr705, p-STAT3Ser727 proteins ↓  **Hippocampus and white matter**  ·MBP ↑  ·Iba-1 positive cells, GFAP-positive cells ↓ | - | Bang et al. 2019 |
| Honokiol | SD rat (6 weeks/male)  ·Two vessel occlusion  ·1 mg/kg for 21 days (intraperitoneal) | **Morris water maze test**  ·Escape latency ↓  ·Swimming time in the target quadrant ↑ | **Hippocampus**  ·NeuN-positive cells, neuronal synaptic spines ↑  ·PSD95 protein ↑  ·AcSOD2/ SOD2, 4-HNE, pSTAT3/STAT3, STAT3, C3d proteins ↓ | **Primary astrocytes from brain of postnatal SD rats** (cobalt chloride-induced chronic hypoxia produced A1 polarization)  ·GFAP, C3d protein ↓  ·C3, Serping1 proteins ↓  ·p-STAT3 protein ↓  ·AcSOD2/SOD2, 4-HNE, mitochondrial ROS ↓ | Hu et al. 2023 |
| Cornel iridoid glycoside (morroniside and loganin = 2.3:1; from *Cornus officinalis* Sieb. et Zucc.) | SD rat (-/male)  ·Bilateral common carotid artery ligation  ·60 and 120 mg/kg/d for a month (intragastric) | - | **Cortex and hippocampus**  ·Neurons, nerve fiber arrangement, demyelination recovered  ·Nissl bodies ↑  ·Iba1^+^ microglial cells ↓  ·CD206 protein ↑  ·Calpastatin ↑  ·Cleavage of spectrin αII protein ↓ | **BV2 cell** (LPS and IFN-γ-stimulated)  ·CCL5, IL- 5, IL-6, IL-1β ↓  ·IL-13 ↑  ·Calpain, NLRP3, ASC, caspase-1, procaspase-1 proteins ↓ | Zheng et al. 2022 |
| Ligustilide (from *Angelica*) | SD rat (8 weeks/male)  ·Bilateral common carotid artery occlusion  ·20 and 40 mg/kg/d for 4 weeks (oral) | **Morris water maze test**  ·Escape latency ↓  ·Time spent in the target quadrant ↑  ·Crossing times of the target platform ↑ | **Cortex and hippocampus**  ·Nissl bodies ↑  ·MDA ↓, SOD, GSH-Px ↑  ·Homocysteine level ↓  ·Bax, cleaved caspase-3 proteins ↓, Bcl-2 protein ↑  ·P-AMPK, SIRT1 proteins ↑ | - | Peng et al. 2022 |
|  | SD rat (-/male)  ·Permanent bilateral common carotid artery occlusion  ·80 mg/kg/d for 7 days (oral) | **Morris water maze test**  ·Escape latency ↓  ·Swimming distance ↓  ·Percent time in the target quadrant ↑ | **Cortex and hippocampus CA1**  ·Neuronal loss and atrophy recovered  ·Nissl staining cells ↑  ·Caspase 3 positive cells ↓  ·MAP-2 labeled dendritic integrity destruction ↓  ·GFAP labeled astrocytes ↓ | - | Feng et al. 2012 |
| Puerarin (from *Pueraria lobata*) | Wistar rats (-/male)  ·Permanent bilateral common carotid artery occlusion  ·50, 100, and 150 mg/kg/d for 5 weeks (intragastric) | **Morris water maze test**  ·Escape latency ↓  ·Crossing times of the target platform ↑  ·Time in the target quadrant ↑ | **Hippocampal CA1 region**  ·Nerve cells arrangement, tissue and neuronal cell loss, damage of neuronal and synaptic structure recovered  ·TUNEL positive cells ↓  ·Bax protein ↓, Bcl-2 protein ↑, Bax/Bcl-2 ↓  ·ROS, MDA ↓, SOD ↑  ·TRPM2 protein ↓, NR2A protein ↑ | **Middle brain artery**  ·Acetylcholine-associated endothelium-dependent relaxation ↑ | Zhu et al. 2021 |
|  | SD rat (2-3 months/male)  ·Permanent bilateral common carotid artery occlusion  ·100 mg/kg/d for 17 days (intraperitoneal) | **Morris water maze test**  ·Escape latency ↓  ·Crossing times of the target platform ↑ | **Hippocampus**  ·Neuronal arrangement, shape recovered  ·pMeCP2 positive cells ↑  ·pMeCP2 protein ↑ | **-** | Wang, Zhang, et al. 2018 |
| Ginsenoside Rg1 (from *Panax ginseng* C.A.Meyer | C57BL/6J mouse (3 months/male)  ·Bilateral common carotid artery stenosis  ·0.1, 1, and 10 mg/kg/d for 14 days (intraperitoneal) | **Morris water maze test**  ·Time in the platform quadrant ↑ | **Hippocampus**  ·Neuronal loss, neuronal apoptosis ↓  ·Apoptotic cells ↓  ·GPR30 mRNA and protein ↑  ·Bax/Bcl-2 ↓ | - | Shen et al. 2021 |
| Ginsenoside Rd  (from *Panax ginseng* C.A.Meyer | C57BL/6J mouse (5-7 weeks/male)  ·Bilateral common carotid artery stenosis  ·10 and 30 mg/kg/d for 21 days (intraperitoneal) | **Morris water maze test**  ·Escape latency ↓  ·Crossing times of the target quadrant ↑  ·Time in the target quadrant ↑  ·Distance in target quadrant ↑ | **Hippocampus and prefrontal cortex**  ·Normal cells ↑  ·BDNF mRNA ↑  ·p300/CBP proteins ↑  ·HDAC2 protein ↓ | **Primary neuronal cell** (OGD/R injury)  ·Cell apoptosis, caspase-3 ↓  ·LDH ↓  ·Histone H3 acetylation ↑  ·HDAC2 protein ↓ | Wan et al. 2017 |
| Curcumin (from *Curcuma longa* L.) | SD rat (six weeks/male)  ·High-fat diet + streptozotocin + bilateral common carotid artery occlusion  ·50 mg/kg/2d for 8 weeks (intraperitoneal) | **Morris water maze test**  ·Escape latency ↓  ·Crossing times of the target platform ↑  ·Time percentage in the target quadrant ↑ | **Hippocampus**  ·Abnormal morphologies of shrunken neurons, neuronal loss recovered  ·Number of neurons ↑  ·Microglial overactivation, density ↓  ·Iba1-positive cells ↓  ·TUNEL-positive cells ↓  ·IL-1β, IL-6, TNF-α mRNAs ↓  ·IL-4, IL-10, Arg-1 mRNAs ↑  ·TREM2 mRNA and protein ↑  ·TLR4 mRNA and protein ↓  ·NF-κB p65 protein ↓  ·Bax mRNA and protein ↓, cleaved caspase-3 mRNA and protein ↓, Bcl-2 mRNA and protein ↑  ·NLRP3, ASC, cleaved-caspase-1, GSDMD-N, IL-18 proteins ↓  ·NRLP3, IL-18 mRNAs ↓ | **BV2 microglial cells** (high glucose + hypoxia induced)  ·Apoptotic cells ↓  ·IL-1β, IL-6, TNF-α mRNAs ↓  ·iNOS, COX-2 mRNAs ↓  ·Arg-1 mRNA ↑  ·TREM2 mRNA and protein ↑  ·TLR4 mRNA and protein ↓  ·NF-κB p65 protein ↓  ·Bax mRNA and protein ↓, cleaved caspase-3 mRNA and protein ↓, Bcl-2 mRNA and protein ↑  ·NLRP3, ASC, cleaved-caspase-1, GSDMD-N, IL-18 proteins ↓  ·NRLP3, IL-18 mRNAs ↓ | Zheng et al. 2021 |
| Salidroside (from *Rhodiola rosea*) | SD rat (2 months/male)  ·Bilateral common carotid artery occlusion  ·30 mg/kg/d for 8 weeks (oral) | **Morris water maze test**  ·Escape latency ↓  ·Time in the target quadrant ↑  ·Time spent in on the platform ↑ | **Hippocampus**  ·MDA ↓, SOD ↑  ·p-p38, caspase-3 proteins ↓ | - | Tian et al. 2020 |
|  | SD rat (-/male)  ·Permanent bilateral common carotid artery occlusion  ·20 mg/kg/d for 34 days (intraperitoneal) | **Morris water maze test**  ·Escape latency ↓  ·Time in the target quadrant ↑ | **Hippocampal** **CA1 region**  ·Anti-NeuN ↑  ·fEPSP ↑  ·Caspase-3, Bax/Bcl-2 ratio proteins ↓ | - | Yan et al. 2015 |
| Osthole (from *Cnidium monnieri* (L.) Cusson) | SD rat (-/male)  ·Modiﬁed bilateral common carotid artery occlusion  ·5, 10, and 20 mg/kg/d for 62 days (oral) | **Morris water maze test**  ·Escape latency ↓  **Y-maze test**  ·Spontaneous alteration ↑ | **Hippocampal** **CA3 region**  ·Neuronal damage recovered  ·Nissl positive cells ↑  ·Activated microglia ↓  ·NLRP3 protein ↓  ·Aβ_1–40_, Aβ_1–42_ proteins ↓  ·APP, BACE1 proteins ↓ | - | Liu, Chen, et al. 2020 |
|  | Wistar rat (9 weeks/-)  ·Permanent occlusion of bilateral common carotid arteries  ·10 and 20 mg/kg/d for 26 days (oral) | **Morris water maze test**  ·Escape latency ↓  ·Time in the target quadrant ↑ | **Hippocampus**  ·Damage of neuronal structure recovered  ·Surviving pyramid cells ↑  ·MDA ↓, GPx, catalase ↑  ·Bax/Bcl-2, active-caspase-3 proteins ↓ | - | Ji et al. 2010 |
| Glycyrrhizic acid (from *Glycyrrhiza glabra*) | SD rat (4-6 months/male)  ·Permanent bilateral common carotid artery occlusion  ·20 mg/kg/d for 30 days (intraperitoneal) | **Novel object recognition test**  ·Discriminative index ↑  **T-maze test**  ·Spontaneous alternation ↑  **Hole board test**  ·Exploratory drive in hole board ↑ | **Hippocampus and white matter**  ·Morphological damages in hippocampal pyramidal neurons restored  ·Hippocampal pyramidal neurons, myelin density ↑  ·Degenerative changes of dendritic spine recovered  ·Number of dendritic spine ↑  ·Oxidized NADH, Cyto-c ↑  ·MDA, protein carbonyls ↓  ·Catalase, SOD, glutathione peroxidase, glutathione reductase, glutathione s-transferase, reduced glutathione ↑  ·pAKT, pGSK 3b proteins ↑  ·p-Tau protein ↓  ·Bax, Bax/Bcl-2, caspase-3, cytosolic cyto-c proteins ↓  ·Bcl-2, mitochondrial cyto-c proteins ↑  ·Cytosolic p-Nrf2 protein ↓  ·Nuclear p-Nrf2, NQO-1, gamma-GCS, HO-1 proteins ↑ | - | Sathyamoorthy et al. 2020 |
|  | Wistar rats (6 weeks/male)  ·Permanent occlusion of the bilateral common carotid arteries  ·20 mg/kg/d for 5 days (oral) | **Morris water maze test**  ·Escape latency ↓  ·Time in the target quadrant ↑ | **Stratum radiatum**  ·EPSP ↑  **Hippocampal CA1 and cerebral cortex**  ·Neuronal cell loss, shrinkage, marked vacuolar changes recovered  ·MDA ↓, SOD ↑  ·Voltage-gated sodium channels and sodium current in pyramidal neurons ↓ | **-** | Guo et al. 2016 |
|  | Wistar rats (-/male)  ·Permanent occlusion of the bilateral common carotid arteries  ·20 mg/kg for 12 days (intraperitoneal) | **Morris water maze test**  ·Escape latency ↓  ·Time in the platform quadrant ↑ | **Cerebral cortex and hippocampus**  ·Pyramidal cellular nuclei shrank, irregular pyramidal layer, cellular edema recovered  ·MDA ↓, SOD ↑ | **PC12 cells** (glutamate-induced neurotoxicity)  ·Bax mRNA ↓, Bcl-2 mRNA ↑ | Hou et al. 2010 |
| Rehmannioside A (from Rehmanniae Radix) | SD rat (8 weeks/male)  ·Permanent occlusion of the bilateral common carotid artery  ·40 and 80 mg/kg/d for 14 days (intraperitoneal) | **Morris water maze test**  ·Escape latency ↓  ·Time in the target quadrant ↑  ·Crossing times of the target platform ↑  ·fEPSP ↑  **Novel object recognition test**  ·Recognition between the familiar and the novel object ↑ | **Hippocampus CA1, CA3 areas**  ·Number of neurons ↑  ·Noradrenaline, dopamine ↓, serotonin ↑  ·AchE ↓, Ach ↑  ·IB4 ↓  ·GSH, eNOS ↑, MDA ↓  ·TNF-α, IL-1β, IL-6 proteins ↓  ·HO-1, Nrf-2 proteins ↑  ·GFAP, p-NF-κB proteins ↓  ·TUNEL, active caspase-3 proteins ↓  ·Cleaved caspase-3, PARP, Cyto-c proteins ↓ | **SH-SY5Y** (hydrogen peroxide-stimulated)  ·ROS cells ↓  ·Apoptotic cells ↓  ·HO-1, Nrf-2 proteins ↑  ·TNF-α, IL-1β, IL-6 mRNAs ↓  ·GFAP, p-NF-κB proteins ↓  ·Cleaved caspase-3, PARP, Cyto-c proteins ↓  ·Cyto-c expressing cells ↓ | Sun et al. 2019 |
| Andrographolide (from *Andrographis paniculata*) | SD rat (-/male)  ·Ligation of bilateral common carotid arteries  ·1% injection 1 mL/kg/d for 4 weeks (intraperitoneal) | **Morris water maze test**  ·Escape latency ↓  ·Crossing times of the target platform ↑  ·Time in the target quadrant ↑ | **Hippocampal CA1**  ·Pyramidal neurons ↑, pyknotic neurons ↓  ·GFAP-positive cells ↓  ·GFAP protein ↓  ·BDNF, TrkB proteins ↑  ·TNF-α, IL-1β proteins ↓  ·Caspase-3 protein ↓ | - | Wang et al. 2019 |
| Scutellarin (from *Scutellaria* spp.) | SD rat (-/male)  ·Permanent bilateral common carotid artery occlusion  ·10 and 30 mg/kg/d for 28 days (oral) | **Morris water maze test**  ·Escape latency ↓  ·Sectional swimming time ↑  ·Memory scores ↑  ·Number of target heading to the platform site ↑  ·First target heading ↓ | **Cerebral cortex and the hippocampus**  ·Aβ_1-40_, Aβ_1-42_ proteins ↓  ·APP, BACE-1 proteins ↓  ·GFAP, Iba1 proteins ↓  ·Number and cell body size of Iba1-expressing microglia ↓ | - | Shin et al. 2018 |
| Ligustrazine (from *Ligusticum chuanxiong* Hort.) | Wistar rat (8 weeks/male)  ·Bilateral common carotid artery occlusion  ·20 and 60 mg/kg/d for 6 weeks (intragastric) | - | **Hippocampus**  ·BDNF ↑, MCP-1, Hcy ↓ | **PC12 cells** (OGD induced)  ·Cellular swelling or membrane integrity loss, necrotic cells, degree of apoptosis ↓  ·BDNF ↑, MCP-1, Hcy ↓  ·Bax/Bcl-2, cleaved caspase-3 proteins ↓ | Zhao et al. 2018 |
| Salvianolic acid B (from *Salvia miltiorrhiza*) | SD rat (2 months/male)  ·Permanent bilateral common carotid artery occlusion  ·20 mg/kg/d for 6 weeks (oral) | **Morris water maze test**  ·Escape latency ↓  ·Crossing times of the target platform ↑ | **Hippocampus**  ·Neuronal structure and arrangement recovered in CA1 region  ·Cell apoptosis ↓  ·IGF-1, p-Akt proteins ↑  ·IGF-1 mRNA ↑ | - | Ma et al. 2017 |
| Tanshinone IIA | SD rat (-/male)  ·Permanent occlusion of bilateral vertebral arteries and bilateral common carotid arteries  ·30 mg/kg/d for 14 days (intravenous) | **Morris water maze test**  ·Escape latency ↓  ·Time in the target quadrant ↑  ·Crossing times of the target platform ↑ | **Hippocampus**  ·TUNEL positive cells in CA1 region ↓  ·p-GSK 3β protein ↑, p-Tau protein ↓  ·ACh, ChAT ↑, AChE,↓ | - | Kong et al. 2017 |
| Tanshinol | SD rat (16-18 months/male)  ·Occlusion of bilateral common carotid arteries  ·15 and 30 mg/kg/d for 28 days (-) | **Water maze test**  ·Swimming distance ↓ | **Hippocampus**  ·IL-1β, IL-6, TNF-α proteins ↓ | **Hippocampal primary neuron** (LPS-induced cytotoxicity)  ·Cell survivals ↑  ·Neuronal apoptosis ↓  ·IL-1β, IL-6, TNF-α mRNAs ↓  ·IL-1β, IL-6, TNF-α, MST1, p-FOXO3 proteins ↓  **Plasma**  ·IL-1β, IL-6, TNF-α ↓ | Yang et al. 2016 |
| Tetrandrine (from *Stephania tetrandra* S. Moore) | SD rat (4 weeks/male)  ·Occlusion of common carotid arteries  ·10 and 30 mg/kg/2d for 4 weeks (intraperitoneal) | **Morris water maze test**  ·Latency for finding the hidden platform ↓  ·Time in the target quadrant ↑ | **Hippocampus CA1 region**  ·Living nerve cells, Nissl granules ↑  ·NR2B-pTyr1472 ↓ | - | Lv et al. 2016 |
|  | SD rat (-/male)  ·Ligation of the bilateral common carotid arteries  ·10 and 30 mg/kg/3d for 4 weeks (intraperitoneal) | **Morris water maze test**  ·Escape latency ↓  ·Swimming distance ↑ | **Hippocampal CA1 region**  ·Neuronal damages recovered  ·Number of cells, Nissl bodies ↑  ·S100B, IL-1β, TNF-α, iNOS proteins and mRNAs ↓ | - | Chen et al. 2013 |
| Betaine | SD rat (12-16 weeks/male)  ·Permanent ligation of bilateral common carotid arteries  ·12.5 mg/kg/d for 14 days (oral) | **Morris water maze test**  ·Escape latency ↓  ·Time in the target quadrant ↑ | **Hippocampus**  ·PSD93, PSD95, MAP2 proteins ↑  ·SOD, GSH ↑, ROS, MDA ↓ | - | Nie et al. 2016 |
| Paeoniflorn (from Paeoniae radix) | SD rat (16-18 months/male)  ·Bilateral common carotid artery occlusion  ·20 and 40 mg/kg/d for 28 days (oral) | **Morris water maze test**  ·Escape latency ↓  ·Distance to hidden platform ↑ | **Hippocampus**  ·NSE, S100β proteins ↓  ·Bcl-2 protein ↑, Bax, cyto-c proteins ↓  ·BDNF protein ↑ | - | Zhang et al. 2016 |
| Icariin (from *Epimedium brevicornum*) | SD rat (10 weeks/male)  ·Permanent bilateral common carotid artery occlusion  ·10 and 40 mg/kg/twice a day for 23 days (intragastric) | **Morris water maze test**  ·Prolongation of latency ↓  ·Swimming distance ↓  ·Time in the target quadrant ↑ | **Hippocampal CA1, CA3**  ·Neuronal damages recovered  ·Aβ_1–40/42_, APP, BACE1 proteins ↓  ·ADAM10, IDE proteins ↑  ·TGF-β1, p-Smad2, p-Smad3 proteins ↓ | - | Li et al. 2015 |
|  | Wistar rat (8-12 weeks/male)  ·Permanent bilateral ligation of the common carotid arteries  ·30, 60, and 120 mg/kg/d for 3 months (-) | **Morris water maze test**  ·Escape latency ↓  ·Searching distance ↓  ·Explore time, distance of the target area ↑ | **Hippocampus**  ·ChAT, AChE proteins ↑  ·MDA ↓, SOD, AChE ↑ | - | Xu et al. 2009 |
| Bilobalide (from *Ginkgo biloba*) | SD rat (2-3 months/male)  ·Bilateral common carotid artery occlusion  ·2, 4, and 8 mg/kg for 60 days (intragastric) | **Morris water maze test**  ·Escape latency ↓  ·Swimming time in the quadrant of platform ↑  ·Number of rats crossing the platform site ↑ | **Cortex and hippocampal CA1 region**  ·Neuronal degeneration recovered  ·Neuronal apoptosis ↓  ·MDA, NOS ↓, SOD, GSH ↑  ·TNF-α immunoreactive cells ↓ | - | Li et al. 2013 |
| Polydatin (from *Polygonum cuspidatum* Sieb. et Zucc.) | SD rat (-/male)  ·Occlusion of vertebral artery and common carotid arteries  ·12.5, 25, and 50 mg/kg/d for 30 days (oral) | **Morris water maze test**  ·Escape latency ↓ | **Hippocampus**  ·MDA ↓, SOD, CAT ↑ | **Cortical neuron** (OGD induced)  ·Cellular body damage, swelling, cellular debris ↓ | Li et al. 2012 |
| Crocin  (from *Crocus sativus* L.) | Wistar rat (-/male)  ·Permanent occlusion of the common carotid arteries  ·0, 10, and 25 mg/kg/day for 5 days (intraperitoneal) | **Morris water maze test**  ·Escape latency ↓  ·Swimming time in the target quadrant ↑  ·Traveled distance to platform ↓ | - | - | Hosseinzadeh et al. 2012 |

4-HNE, 4-hydroxynonenal; ACh, acetylcholine; AChE, acetylcholinesterase; AcSOD, acetylated superoxide dismutase; ADAM10, a disintegrin and metalloprotease 10; AKT, protein kinase B; AMPK, AMP-activated protein kinase; APP, amyloid precursor protein; ASC, apoptosis-associated speck-like protein containing a caspase recruitment domain; ATP5A, adenosine triphosphate synthase F1 subunit alpha; BACE1, β-site APP-cleaving enzyme 1; Bax, Bcl-2 associated X protein; Bcl-2, B cell lymphoma-2; BDNF, brain-derived neurotrophic factor; CaMKII-α, calcium/calmodulin-dependent protein kinase II; CAT, catalase; CBP, CREB-binding protein; CCL5, chemokine (C-C motif) ligand 5; ChAT, choline acetyltransferase; COX, cyclooxygenase; CREB, cAMP response element-binding protein; Cyto-c, cytochrome c; Drp1, dynamin-related protein 1; eNOS, endothelial nitric oxide synthase; ERK1/2, extracellular Signal-Regulated Kinase 1/2; fEPSP, field excitatory postsynaptic potential; Fis1, mitochondrial fission 1 protein; FOXO3, forkhead box protein O3; GCS, gamma-glutamylcysteine synthetase; GFAP, glial fibrillary acidic protein; GLUT, glucose transporter; GPR30, G protein-coupled estrogen receptor 1; GPX4, glutathione peroxidase 4; GSDMD-N, gasdermin D N-terminal fragment; GSH, glutathione; GSK-3β, glycogen synthase kinase-3β; Hcy, homocysteine; HDAC2, histone deacetylase 2; HIF-1, hypoxia-inducible factor-1; HO-1, heme oxygenase 1; HUMMR, hypoxia-upregulated mitochondrial movement regulator; IDE, insulin degrading enzyme; IGF-1, insulin-like growth factor-1; iNOS, inducible nitric oxide synthase; IL, interleukin; IB4, isolectin-B4; Iba1, ionized calcium binding adapter molecule 1; IDE, insulin-degrading enzyme; Keap1, Kelch-like ECH associated protein 1; LC3, microtubule-associated protein 1A/1B-light chain 3; LDH, lactate dehydrogenase; MAP-2, microtubule-associated protein-2; MAPK, mitogen-activated protein kinase; MBP, myelin basic protein; MDA, malondialdehyde; MCP-1, monocyte chemoattractant protein-1; MECP2, methyl CpG binding protein 2; Mfn1/2, mitofusins 1,2; MMP, Mitochondrial membrane potential; MST1, mammalian Ste20-like kinase 1; MTCO1, mitochondrially encoded cytochrome c oxidase subunit 1; mtROS, mitochondrial reactive oxygen species; MYD88, myeloid differentiation primary response 88; NADH, nicotinamide adenine dinucleotide hydrogen; NDUFB8, ubiquinone Oxidoreductase Subunit B8; NeuN, neuronal nuclei; NF-κB, nuclear factor kappa B; NMDAR, N-methyl-d-aspartic acid receptor 1; NLRP3, nucleotide oligomerization domain-like receptors 3; NO, nitric oxide; NQO1, NAD(P)H:quinone oxidoreductase 1; NR2A, N-methyl-D-aspartate receptor 2A; NR2B-pTyr1472, N-methyl-D-aspartate receptor 2B phosphorylation at tyrosine 1472; Nrf2, nuclear factor-E2-related factor 2; NSE, neuron‑specific enolase; OGD/R, oxygen-glucose deprivation/reperfusion; P2X7R, purinergic P2X receptor 7; p300, E1A-binding protein p300; PARP, poly(ADP-ribose) polymerase; PI3K, phosphatidylinositol-3-kinase; PRDX1, peroxiredoxin-1; PSD-95, postsynaptic density protein 95; ROS, reactive oxygen species; S100B, S100 calcium-binding protein B; SDHB, succinate dehydrogenase complex iron sulfur subunit B; SIRT3, Sirtuin 3; SMAD, suppressor of mothers against decapentaplegic; SOD, superoxide dismutase; STAT, signal transducers and activators of transcription; SYP, synaptophysin; TFAM, transcription factor A, mitochondrial; TGF-β, transforming growth factor-β; TLR4, toll-like receptor 4; TNF, tumor necrosis factor; TRAK2, trafficking protein kinesin 2; TREM2, triggering receptor expressed on myeloid cells 2; TrkB, tropomyosin receptor kinase B; TRPM, transient receptor potential melastatin; UQCRC2, ubiquinol-cytochrome c reductase core protein 2; VAChT, vesicular acetylcholine transporter.

Table S5 summarizes major rodent models used to induce vascular cognitive impairment and dementia. For each model we list species, method of hypoperfusion or risk-factor induction, typical hemodynamic and histopathological changes, behavioral readouts, and key advantages or limitations.

**Table S5**. Overview of common rodent models of VaD.

| **Model** | **Species** | **Intervention** | **CBF Change** | **Key Pathology** | **Behavioral Readouts** | **Advantages** | **Limitations** |
| --- | --- | --- | --- | --- | --- | --- | --- |
| BCCAo (2VO) | Rat | Permanent ligation of both CCAs | ↓↓ to <20% immediately | WM vacuolation; BBB disruption; gliosis; variable hippocampal neuron loss | Morris water maze deficits by 7 d; radial-arm maze errors | Global hypoperfusion; robust WM injury | High lesion variability; optic tract damage; limited genetics |
| 4VO | Rat | Permanent occlusion of both CCAs + VAs (staged) | ↓↓ to <20% immediately | Cortical & hippocampal neuronal death; gliosis | Aversive radial maze impairment; anxiety in open field | Consistent global ischemia; minimal seizures | Complex two- or three-stage surgery; strain differences |
| UCCAo | Mouse | Permanent ligation of one CCA | ↓ to ~60% ipsilateral | Delayed corpus callosum WM lesions; microgliosis | Novel object recognition loss; elevated plus-maze anxiety | Single-vessel model; mild mortality | No discrete infarcts; contralateral compensation |
| BCAS | Mouse | Bilateral CCA stenosis with microcoils (ID0.18 mm) | Acute ↓30–40%; partial recovery to ~80% | CC WM rarefaction; gliosis; delayed hippocampal atrophy | Y-maze working-memory deficits by 28 d; rotarod motor loss | High reproducibility; transgenic compatibility | Initial ischemic phase; partial CBF recovery |
| GCAS | Mouse | Bilateral CCA stenosis via ameroid constrictors | Gradual ↓ to ~50% over 28 d | Intimal thickening; CC WM gliosis; oligodendrocyte loss | Y-maze & rotarod deficits by 28 d; preserved reference memory | Truly progressive hypoperfusion; atherosclerosis-like stenosis | Requires custom ameroids; surgical expertise |
| SHRSP | Rat | Spontaneous hypertension (genetic) | ↓ variable with age | WM rarefaction; microbleeds; vascular wall thickening | Morris water maze; novel object recognition deficits | Models of chronic hypertension + stroke risk | Frequent spontaneous strokes; behavioral variability |
| DOCA-salt hypertension | Rat | DOCA injections + high-salt diet | ↑ arterial pressure; ↓ CBF | Endothelial dysfunction; oxidative stress | Open field hyperactivity; passive avoidance learning loss | Non-invasive BP elevation | Requires mineralocorticoid dosing; electrolyte imbalance |
| STZ-diabetes | Mouse or rat | Streptozotocin injection (single/high-dose) | Secondary ↓ CBF | Microvascular damage; neural apoptosis | Y-maze, Barnes maze deficits; increased anxiety | Models diabetic vasculopathy; BBB breakdown | High systemic toxicity; strict glucose monitoring |
| HHcy (L-methionine) | Mouse | Dietary L-methionine supplementation | ↓ modestly | Endothelial dysfunction; oxidative stress | Morris water maze deficits; T-maze errors | Simple diet induction; reversible with B-vitamins | High mortality at severe levels; off-target metabolic effects |
| CADASIL transgenic (NOTCH3 R169C mouse) | Mouse | Humanized NOTCH3 mutation | ↓ CBF by ~20%–30% | Leptomeningeal arteriopathy; WM lesions | Y-maze working-memory loss; attentional impairments | Genetic model of pure small-vessel arteriopathy | Late-onset pathology; specialized breeding |
| CAA transgenic (e.g. Tg-SwDI) | Mouse | Human APP Dutch/Iowa mutation | ↓ variable | Vascular amyloid deposition; microhemorrhages | Contextual fear conditioning deficits; gait changes | Reproduces amyloid angiopathy and microbleeds | Mixed AD/VaD pathology; slower onset |
| Multiple-microinfarct (microspheres) | Rat | Injection of 48–50 µm microspheres into ICA | ↓ focal WM & cortex | Numerous small infarcts in cortex, striatum, hippocampus | Active/passive avoidance deficits; water maze errors | Models patchy lacunar infarcts | Low reproducibility; infarct number & location variable |
| Cholesterol-crystal microinfarcts | Mouse | ICA injection of 40–70 µm cholesterol crystals | ↓ focal | Cortical & subcortical microinfarcts; gliosis | Novel object recognition; fear-conditioning deficits | Clinically relevant lipid emboli model | Inflammatory dominated; lesion coalescence over time |
| High-fat diet | Mouse | 40–60% kcal from fat for ≥3 months | ↓ modestly (~10–20%) | Hippocampal synaptic loss; neuroinflammation | Morris water maze; novel object deficits | Models obesity-driven vascular inflammation | Requires long feeding; variable WM pathology |

BBB, blood–brain barrier; CBF, cerebral blood flow; CCA, common carotid artery; ICA, internal carotid artery; d, days; WM, white matter.

In our primary analysis, we focused on BCAS and BCCAo because they are the two most commonly employed methods to induce chronic cerebral hypoperfusion in rodents. Below we compare their key features, pathological hallmarks, behavioral readouts, and practical considerations. Both models reproduce key features of human subcortical ischemic vascular dementia but differ in hemodynamic profile, lesion distribution, genetic compatibility, and behavioral endpoints. Researchers should select BCAS for transgenic and WM-focused studies, and BCCAo for global hypoperfusion and infarct‐driven paradigms.

**Table S6**. Summary of the two most frequently used rodent models of VaD

| **Model** | **Species** | **Intervention** | **CBF Change** | **Histopathology** | **Behavioral Phenotype** | **Advantages** | **Limitations** |
| --- | --- | --- | --- | --- | --- | --- | --- |
| BCAS (bilateral common-carotid-artery stenosis) | Mouse (C57BL/6J) | Steel microcoils (ID ≈0.18 mm) placed around each CCA, 30 min apart | Acute drop to ≈60-70% within hours  Partial recovery to ≈80% by 28 d | White-matter rarefaction & demyelination (corpus callosum)  Astro-/microgliosis  Minimal early hippocampal loss | Working-memory deficit (Y-maze) by 28 d  Motor impairment (rotarod) by 28 d  Reference memory preserved until ≈6 months | High reproducibility  Applicable to transgenic lines  Moderate mortality (≈10-20%) | Acute “ischemic” phase  Partial CBF recovery  No single-model microinfarcts |
| BCCAo (bilateral CCA occlusion, “2VO”) | Rat (Wistar) | Permanent ligation of both CCAs under pentobarbital anesthesia | Near-complete (>80%) drop immediately  Stable over weeks | Widespread white-matter vacuolation  BBB disruption  Hippocampal CA1/CA3 neuronal loss (variable) | Spatial-memory deficits (Morris water maze) by 7 d  Impaired radial-arm maze performance | Stable, global hypoperfusion  Robust WM injury | High lesion variability  Visual-pathway damage confounds tasks  Not readily applied to most transgenic strains |

BBB, blood–brain barrier; CBF, cerebral blood flow; CCA, common carotid artery; ID, inner diameter; d, days; WM, white matter.

**Table S7**. Network group of herbal ingredients

| Herb ingredient | Group |
| --- | --- |
| Alismatis Rhizoma | 1 |
| Angelicae Sinensis Radix | 1 |
| Atractylodis Rhizoma Alba | 1 |
| Carthami Flos | 1 |
| Chuanxiong Rhizoma | 1 |
| Moschus | 1 |
| Paeoniae Radix | 1 |
| Persicae Semen | 1 |
| Poria Sclerotium | 1 |
| Rehmanniae Radix Praeparata | 1 |
| Acori Tatarinowii Rhizoma | 2 |
| Alpiniae Oxyphyllae Fructus | 2 |
| Curcumae Radix | 2 |
| Lycii Fructus | 2 |
| Notoginseng Radix et Rhizoma | 2 |
| Polygalae Radix | 2 |
| Polygoni Multiflori Radix | 2 |
| Salviae Miltiorrhizae Radix et Rhizoma | 2 |
| Schisandrae Fructus | 2 |
| Astragali Radix | 3 |
| Ganoderma | 3 |
| Hirudo | 3 |
| Pheretima | 3 |
| Puerariae Lobatae Radix | 3 |
| Bupleuri Radix | 4 |
| Citri Unshius Pericarpium | 4 |
| Gastrodiae Rhizoma | 4 |
| Ginseng Radix | 4 |
| Glycyrrhizae Radix et Rhizoma | 4 |
| Morindae Officinalis Radix | 4 |
| Ophiopogonis Radix | 4 |
| Pinelliae Tuber | 4 |
| Rhei Radix et Rhizoma | 4 |
| Uncariae Ramulus cum Uncus | 4 |
| Zingiberis Rhizoma | 4 |
| Modularity | 0.336057 |

**Table S8**. Usage frequency (≥ 5) of herbal ingredients occurring in the herbal formulas

| Herb ingredient | Frequency |
| --- | --- |
| Chuanxiong Rhizoma | 15 |
| Ginseng Radix | 11 |
| Paeoniae Radix | 10 |
| Astragali Radix | 9 |
| Salviae Miltiorrhizae Radix et Rhizoma | 9 |
| Angelicae Sinensis Radix | 9 |
| Pheretima | 7 |
| Poria Sclerotium | 7 |
| Persicae Semen | 6 |
| Glycyrrhizae Radix et Rhizoma | 6 |
| Acori Rhizoma | 6 |
| Puerariae Lobatae Radix | 5 |
| Carthami Flos | 5 |
| Zingiberis Rhizoma (including Zingiberis Rhizoma Recens) | 5 |
| Polygalae Radix | 5 |
| Pinelliae Tuber | 5 |
| Atractylodis Rhizoma (including Atractylodis Rhizoma Alba) | 5 |
| Notoginseng Radix et Rhizoma | 5 |

**References**

S1. Meng, X., Z. Ruan, B. Chen, et al. 2020. “Wuzang Wenyang Huayu decoction regulates differentially expressed transcripts in the rats' hippocampus after cerebral hypoperfusion.” *Journal of Cellular and Molecular Medicine* 24: 294–303. https://doi: 10.1111/jcmm.14723.

S2. Han, H. S., J. H. Jang, J. S. Choi, et al. 2010. “Water extract of *Triticum aestivum* L. and its components demonstrate protective effect in a model of vascular dementia.” *Journal of Medicinal Food* 13: 572–578. https://doi:10.1089/jmf.2009.1242.
